# A Data Similarity-Based Strategy for Meta-analysis of Transcriptional Profiles in Cancer

**DOI:** 10.1371/journal.pone.0054979

**Published:** 2013-01-29

**Authors:** Qingchao Qiu, Pengcheng Lu, Yuzhu Xiang, Yu Shyr, Xi Chen, Brian David Lehmann, Daniel Joseph Viox, Alfred L. George, Yajun Yi

**Affiliations:** 1 Department of Medicine, Vanderbilt University, Nashville, Tennessee, United States of America; 2 Cancer Research Institute and Human Morphology Center, University of South China, Hengyang, Hunan, China; 3 Department of Biostatistics, Vanderbilt University, Nashville, Tennessee, United States of America; 4 Minimally Invasive Urology Center, Provincial Hospital Affiliated to Shandong University, Jinan, Shandong, China; 5 Department of Biochemistry, Vanderbilt University, Nashville, Tennessee, United States of America; 6 Feinberg School of Medicine, Northwestern University, Chicago, Illinois, United States of America; 7 Institute for Integrative Genomics, Vanderbilt University, Nashville, Tennessee, United States of America; Harvard School of Public Health, United States of America

## Abstract

**Background:**

Robust transcriptional signatures in cancer can be identified by data similarity-driven meta-analysis of gene expression profiles. An unbiased data integration and interrogation strategy has not previously been available.

**Methods and Findings:**

We implemented and performed a large meta-analysis of breast cancer gene expression profiles from 223 datasets containing 10,581 human breast cancer samples using a novel data similarity-based approach (iterative EXALT). Cancer gene expression signatures extracted from individual datasets were clustered by data similarity and consolidated into a meta-signature with a recurrent and concordant gene expression pattern. A retrospective survival analysis was performed to evaluate the predictive power of a novel meta-signature deduced from transcriptional profiling studies of human breast cancer. Validation cohorts consisting of 6,011 breast cancer patients from 21 different breast cancer datasets and 1,110 patients with other malignancies (lung and prostate cancer) were used to test the robustness of our findings. During the iterative EXALT analysis, 633 signatures were grouped by their data similarity and formed 121 signature clusters. From the 121 signature clusters, we identified a unique meta-signature (BRmet50) based on a cluster of 11 signatures sharing a phenotype related to highly aggressive breast cancer. In patients with breast cancer, there was a significant association between BRmet50 and disease outcome, and the prognostic power of BRmet50 was independent of common clinical and pathologic covariates. Furthermore, the prognostic value of BRmet50 was not specific to breast cancer, as it also predicted survival in prostate and lung cancers.

**Conclusions:**

We have established and implemented a novel data similarity-driven meta-analysis strategy. Using this approach, we identified a transcriptional meta-signature (BRmet50) in breast cancer, and the prognostic performance of BRmet50 was robust and applicable across a wide range of cancer-patient populations.

## Introduction

Breast cancer is the most common type of cancer in women and the second-leading cause of cancer death among women in the United States. A molecular biomarker that can predict the likelihood of cancer progression to invasive or metastatic disease can guide how aggressively patients are initially treated [1]. There is a clear need for a better understanding of how molecular profiles relate to cancer phenotypes and clinical outcomes and for new cancer biomarkers with definable and reproducible performance in diverse patient populations.

The introduction of genome-scale gene expression profiling has led to the identification of specific transcriptional biomarkers known as gene expression signatures. The discovery of gene expression signatures from any single well-powered study is relatively straightforward. Some signatures have utility as transcriptional biomarkers for classifying patients with significantly different survival outcomes in breast cancer [2], [3]. For example, transcriptional profiling of primary breast cancer has been used previously to identify a 70-gene signature (marketed as MammaPrint but designated here as BRsig70) [3], a distinct 76-gene signature (BRsig76) [2], and others (Oncotype DX [4], [5], TAMR13 [6], Genius [7], GGI [8], PAM50 [9] and PIK3CAGS278 [10]). Typical of other transcriptional biomarkers, both BRsig70 and BRsig76 were derived from a training set from a single study and then validated with a test set from the same retrospective patient cohorts. When subjected to external validation, most signatures could only be validated using one dataset (NKI295) [11] or a few smaller datasets with retrospectively accrued samples. This validation method has inevitable limitations of statistical power or sample selection bias. As a result, a common weakness of this approach is its lack of consistency and reproducibility [12]–[16].

With hundreds of breast cancer gene expression datasets deposited in public databases, we now have the ability to utilize these data to their full potential and discover recurrent and reliable gene expression signatures for breast cancer prognosis prediction. However, the identification of a prognostic expression signature through meta-analysis of publicly available cancer gene expression profiles represents an underexploited opportunity. There are several reports of meta-analysis frameworks that use multiple breast cancer datasets to build and validate prognostic classifiers [7], [17], [18]. These approaches focus on selecting predictors from combined training sets, either using average Cox-scores [18] or taking into account the sample molecular subtypes [7], [17]. However, one unanswered question is how to identify homogeneous gene expression studies using a refined and unbiased selection method [19]. In order to extrapolate validated prognostic signatures to a broader patient population, new biostatistical methods using data similarity-based analysis are needed [20].

To avoid the weaknesses of single study-derived signatures and to generate a new strategy to better utilize the available gene expression data from independent studies, we have developed a meta-analysis strategy called EXALT (EXpression AnaLysis Tool) [21], [22]. The essential feature of EXALT is a database containing thousands of gene expression signatures extracted from published studies that enables signature comparisons. In this study, we used EXALT in an iterative manner (iterative EXALT) to conduct a data similarity-driven meta-analysis and elucidate transcriptional signatures with enhanced prognostic value in breast cancer. We demonstrated that heterogeneous signatures from 223 public datasets containing 10,581 breast cancer samples could be systematically organized by their common data elements (i.e., intrinsic similarities and disease phenotypes) and assembled into a new signature data type called a meta-signature. We identified a specific meta-signature consisting of 50 genes (BRmet50) that is robustly predictive of cancer prognosis in 6,011 breast cancer patients from 21 different breast cancer datasets as well as in other malignancies including lung and prostate cancer. These findings illustrate the value of BRmet50 in breast cancer prognosis independent of treatment variables and indicate that iterative EXALT is a novel meta-analysis method capable of performing informative and robust discovery of meta-signatures in cancer.

## Results

### Extraction of Human Cancer Signatures

**Figure 1:**
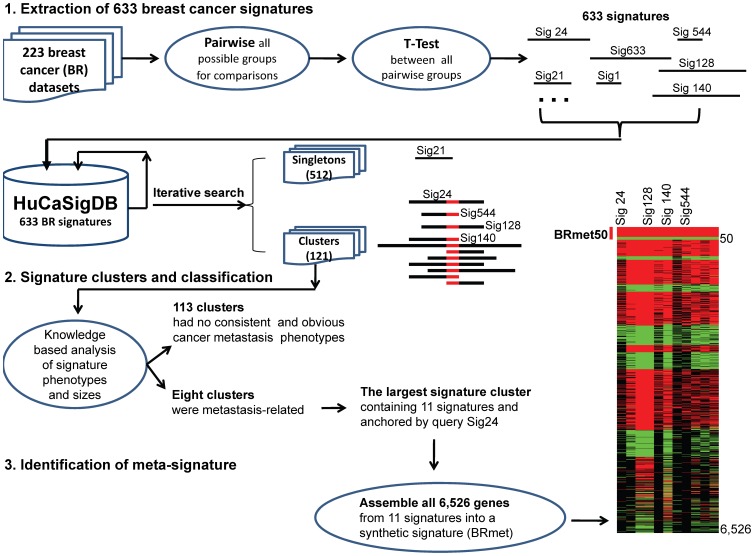
Signature clustering process for identification of BRmet50. The workflow of iterative EXALT method includes three major processes. (1) Extraction of 633 breast cancer signatures. All paired sample groups within each breast cancer datasets (n = 223) were compared based on all possible clinical and pathologic covariates such as tumor size, nodal involvement, grade, marker status, lymphovascular invasion, relapse, metastasis, p53 status, BRCA1 and BRCA2 mutations. Student’s t-test was then performed for all pairwise comparisons, and a total of 633 breast cancer signatures were generated and uploaded into a database (HuCaSigDB). (2) Signature clusters and classification. Iterative search was carried out using each of 633 signatures as a query (anchored or seed) signature against HuCaSigDB repeatedly to identify homologous signatures with significant data similarity defined by EXALT. 121 out of 633 query signatures found at least one similar signature in HuCaSigDB and formed 121 clusters, while the remaining 512 (singletons) failed to generate clusters. Two typical results are depicted by schematic description labeled with anchored signatures: the singleton Sig21 and the cluster Sig24 including 11 signature members like Sig544, Sig128, Sig140, etc. Knowledge based analysis of signature phenotypes and sizes was performed among 121 signature clusters. Eight clusters had obvious metastasis phenotypes. Of the eight clusters, the largest cluster anchored by the query signature (sig24) was selected for further analysis. (3) Identification of meta-signature BRmet50. All 6,526 signature genes from the 11 signatures of the cluster Sig24 were assembled together to form a synthetic signature (BRmet). The genes within BRmet were ranked based on recurrent frequency and concordance of differential expression represented by a meta-heat map. The top 50 genes (BRmet50) represented in rows were determined by a 100% recurrent frequency and gene expression profile concordance among the 11 signatures represented in columns. The colors in the meta-heat map represent the direction of differential gene expression within a given transcriptional profile (red for up, green for down, and black for a missing match). Color intensity reflects the confidence levels of differential expression.

To organize the complex transcriptional data, we have established a hierarchical data structure. The top level consists of the transcriptional studies, and each transcriptional study was partitioned into three levels: data sets, groups, and samples. A study can include one or many data sets depending on its experimental design [21]. From 56 breast cancer studies (Table S1), we have collected 223 breast cancer data sets representing 10,581 breast cancer samples. Primary breast cancer samples within each dataset were grouped by their clinical attributes. Each dataset included at least two groups of tumor samples with various clinical phenotypes (Figure 1 top panel). For example, the phenotypes related to cancer relapse or poor prognosis include tumor size, nodal involvement, grade, lymphovascular invasion, p53 status, BRCA1 mutation, BRCA2 mutation, estrogen receptor (ER), and human epidermal growth factor receptor 2 (HER2) status [23], [24]. Two or more groups per dataset were needed to generate statistical comparisons. A total of 633 significant gene lists (“simple signatures”) from all possible pairwise group comparisons were generated accordingly using a Student’s t-test [21]. All 633 “simple signatures” were then stored in a human cancer signature database (HuCaSigDB) that is accessible online (http://seq.mc.vanderbilt.edu/exalt/) [22]. The major procedural steps for extraction of signatures are provided in the Methods S1.

A gene expression signature (“simple signature”) as defined by EXALT is a set of significant genes with their corresponding statistical scores and gene expression direction codes (up or down). Some “simple signatures” are biologically related to breast cancer prognosis, but they were derived from individual transcription profiling studies and are all too often underpowered, truncated, or of low quality. There are inherent limitations for any individual profiling study including small sample size relative to the large number of potential predictors, limitations of technological platforms, sample variation, and bioinformatics or statistical method bias. An underlying assumption we made in formulating this approach is that any individual transcriptional profiling study does not decode an entire expression signature. Rather, these “simple signatures” represent only fragments of a complete and common transcriptional profile (meta-signature).

### Identification of a Novel Breast Cancer Meta-signature

We hypothesized that a meta-signature with improved predictive power could be discovered by data similarity-driven meta-analysis of transcriptional profiles from multiple related studies. EXALT analysis provided the basis for grouping or clustering “simple signatures” sharing significant data similarity. The iterative EXALT process gathered homologous signatures from “simple signatures” and consolidated them into meta-signatures (Figure 1 middle and lower panel). Briefly, each breast cancer signature was compared with all breast cancer signatures in HuCaSigDB, and signature pairs with significant similarity were grouped together. The intrinsic relationship between pairwise signatures was first determined by gene symbol match and concordance in the direction of gene expression change. Then, a normalized total identity score was calculated based on Q-values from the two signatures. The significant similarity level were determined by simulation analysis [21] as explained in the Methods S1.

We performed iterative EXALT analyses in which all-versus-all signature similarity searches were carried out. More specifically, each of the 633 “simple signatures” from HuCaSigDB served as a seed (also called query or anchored signature) to query all “simple signatures” in HuCaSigDB repeatedly and to bring other homologous signatures together by their common elements (i.e., intrinsic similarities). This iterative process “grouped” or “clustered” signatures based on their similarities (Figure 1 middle panel). Signature pairs that were sufficiently similar (p<0.05) were linked together to form clusters. After iterative comparisons, each seed signature either remained as a singleton (i.e., a seed signature that self-matched but did not match any other signatures) or formed a cluster with other signatures.

This iterative EXALT process starting with 633 seed signatures resulted in 121 signature clusters and 512 singletons (Figure 1 middle panel). We focused on eight specific clusters because the eight seed signatures and all other clustered signatures in each of the eight were clearly related to cancer metastasis. The remaining 113 clusters had no consistent and obvious cancer metastasis phenotypes. For the eight metastasis-related clusters, each contained various overlapping signature members associated with phenotypes that are known risk factors for cancer metastasis such as high-grade tumors, ER-negative status, basal-like cell type, and cancer relapse. Of these, we selected the largest signature cluster containing 11 metastasis-related signatures (Figure 1 and Table 1) [2], [3], [6], [8], [11], [25]–[29]. Because each signature in the cluster was derived from a comparison between highly aggressive and less aggressive breast cancers, this comparison yielded a “poor-prognosis” gene signature (Table 1).

**Table 1:** Members of breast cancer metastatic signature (BRmet50).

Signature ID	Signature Name	BRid*
Sig544	without metastasis vs with metastasis	BR544 [3]
Sig2411	without metastasis vs with metastasis	BR2411 [11]
Sig1405	ER-positive vs ER-negative	BR1405 [2]
Sig1128	grade 1 vs grade 3	BR1128 [25]
Sig1042	grade 1 vs grade 3	BR1042 [8]
Sig1224r	ER-positive vs ER-negative	BR1224 [26]
Sig1552r	normal breast-like vs basal-like	BR1552 [27]
Sig1095	grade 1 vs grade 3	BR1095 [28]
Sig1414	grade 1 vs grade 3	BR1414 [28]
Sig1141	grade 1 vs grade 3	BR1141 [6]
Sig907r	normal breast-like vs basal-like	BR907 [29]

* BRid denotes the breast cancer dataset ID sharing the same signature ID number as the respective published study.

Each of the 11 signatures comprises several hundred genes. In order to identify a recurrent and concordant gene expression pattern in the metastatic signature cluster, all genes that comprised the 11 signatures (n = 6,526) were assembled into a synthetic signature designated as BRmet. The genes within BRmet were ranked based on recurrent frequency and direction of differential expression (meta-direction) among all 11 signatures. A 100% recurrent frequency was applied to select the top 50 genes for the meta-signature (BRmet50) (Figure 1 lower panel). Thus, BRmet50 profiles are concordant among all 11 clustered simple signatures (Table 1). BRmet50 genes represent significantly differentially expressed genes not only within their own datasets but also across 11 other related datasets (Figure 1).

Annotation for BRmet50 genes is provided in Table S3. Only five genes in BRmet50 overlapped with BRsig70, and two were found in common with BRsig76. The number of overlapping genes between BRmet50 and the six other cancer signatures (Oncotype DX, TAMR13, Genius, GGI, PAM50, and PIK3CAGS278) is relatively low (1%−27%), suggesting that BRmet50 is a distinct signature. Because BRmet50 was deduced from a cluster of signatures comparing highly aggressive and less aggressive breast cancers, we predicted that BRmet50 would be associated with poor prognoses in breast cancer such as cancer relapse, metastasis, and death. The general prognosis feature of BRmet50 might be different than those of BRsig70/76 (BRmet70 and BRmet76) because they were designed specifically to predict distant metastasis in early-stage breast cancer patients with lymph node-negative status [2], [3]. Thus, we realized that neither BRsig70 nor BRsig76 was fully comparable to BRmet50. Rather, they served as prognostic control signatures in this study.

### Meta-validation of BRmet50 in Breast Cancer

Since the BRmet50 was deduced from a signature cluster comparing more and less aggressive cancers, we retrospectively examined the ability of BRmet50 to predict prognosis in 21 datasets, including 11 independent validation datasets not used in the signature clustering process (Table 2).

**Table 2:** Summary of survival analysis p-values in breast cancer.

Test Data Sets	Endpoints*	BRmet50	BRmet50 Ctr**	BRSig70	BRSig76
***Training datasets***					
BR544 [3]	DMFS	<0.001	<0.001	0.007	0.024
BR2411 [11]	RFS	<0.001	<0.001	<0.001	<0.001
BR1405 [2]	RFS	0.002	0.002	0.019	0.006
BR1128 [25]	DSS	<0.001	<0.001	0.015	0.018
BR1042 [8]	RFS	0.002	0.033	0.144	0.698
BR1552 [27]	RFS	<0.001	<0.001	0.082	<0.001
BR1095 [28]	DFS	<0.001	<0.001	0.001	0.005
BR1414 [28]	RFS	<0.001	<0.001	<0.001	<0.001
BR1141 [6]	RFS	<0.001	<0.001	0.026	0.156
BR18347175 [90]	DMFS	<0.001	NA***	<0.001	<0.001
***Validation datasets***					
METABRIC discovery [91]	DSS	<0.001	NA	<0.001	<0.001
METABRIC validation [91]	DSS	<0.001	NA	<0.001	<0.001
GSE2607 [89]	RFS	0.004	NA	0.005	<0.001
GSE7390 [88]	RFS	0.028	NA	0.516	0.063
GSE11121 [87]	DMFS	0.027	NA	0.012	0.183
GSE17705 [85]	DMFS	0.045	NA	0.043	0.574
GSE20624 [84]	RFS	0.001	NA	0.037	0.037
GSE20685 [83]	OS	<0.001	NA	0.002	<0.001
GSE21653 [86]	DFS	0.014	NA	0.121	0.396
GSE25055 [82]	DMFS	<0.001	NA	<0.001	<0.001
GSE25065 [82]	DMFS	<0.001	NA	<0.001	<0.001

* Endpoints: Clinic endpoints are distant metastases-free survival (DMFS),relapse-free survival (RFS), disease-free survival (DFS), disease-specific survival (DSS), Overall Survival (OS).

** BRmet50 Ctr: control signatures are isoform signatures of BRmet50 assembled by the leave-one-out method in which the corresponding breast cancer dataset is excluded intentionally.

*** NA: not available.

To examine the stability of the iterative EXALT method and to avoid over-fitting of the nine training datasets, we used a ‘leave-one-out’ cross-validation strategy to deduce nine BRmet50 control signatures for the corresponding nine training datasets. In each leave-one-out trial, the included signatures remained clustered. Furthermore, all BRmet50 control signatures from the ‘leave-one-out’ procedure shared the core set of the 50 genes. We then tested these control meta-signatures in corresponding training datasets (Table S2) and found that their prognostic performances were as good as BRmet50 (Table 2). Data suggest that iterative EXALT-based clustering process is a stable and reliable method that is not affected by any particular signature member in the BRmet cluster.

The 11 independent validation datasets were used to evaluate BRmet50 prognosis performance. Log-rank tests were conducted to assess the differences in survival analysis. The p-values from log-rank tests comparing BRmet50, BRsig70, BRsig76, and the six other published cancer signatures (Oncotype DX, TAMR13, Genius, GGI, PAM50 and PIK3CAGS278) are summarized (Table 2 and Table 3). Each signature was evaluated for its ability to classify subjects with breast cancer into ‘good’ and ‘poor’ prognostic groups. Expression values for each signature were retrieved from each corresponding dataset, then unsupervised hierarchical clustering was performed using the Spearman rank correlation, and group assignments were determined in each dataset based on the first bifurcation of the clustering dendrograms [30]. BRmet50 distinguished between the good and poor prognostic groups successfully in all datasets (Table 2), while BRsig70 and BRsig76 could not discriminate prognosis groups in four and six datasets respectively. The failure of BRsig70 and BRsig76 to stratify prognostic groups in those datasets persisted after we re-classified samples using the original algorithms (e.g., either the Pearson correlation method [3] or the relapse score method based on weighted Cox’s regression coefficient values [2]). Thus, these results were independent of statistical methods. Similar results were also obtained among the six other well-established cancer signatures because none of them could discriminate prognosis groups in all 11 test datasets (Table 3). As another performance measure, we calculated the c-index for the cancer signatures in 11 validation datasets (Table 3), which is a generalization of the area under the receiver operating characteristic (ROC) curve [31]. The prognostic value (c-index) for BRmet50 and the other cancer signatures were compared. For any given test dataset, BRmet50 c-index is similar to those from the other cancer signatures, suggesting that the BRmet50 and other cancer signatures provide comparable prognostic information.

**Table 3:** Summary of survival analysis p-values and c-indexes in breast cancer.

	BRmet50	BRsig70	BRsig76	ONCO	TAMR13	PAM50	Genius	PIK3	GGI
**p-values**									
**METABRIC D**	<0.001	<0.001	<0.001	<0.001	<0.001	<0.001	<0.001	0.001	<0.001
**METABRIC V**	<0.001	<0.001	<0.001	<0.001	<0.001	<0.001	<0.001	0.002	<0.001
**GSE2607**	0.004	0.005	<0.001	0.01	0.814	0.078	0.814	0.814	0.814
**GSE7390**	0.028	0.516	0.063	0.368	0.238	0.223	0.013	0.911	0.015
**GSE11121**	0.027	0.012	0.183	0.002	<0.001	0.004	0.003	0.012	0.122
**GSE17705**	0.045	0.043	0.574	0.064	0.002	0.015	0.858	0.677	0.137
**GSE20624**	0.001	0.037	0.037	0.003	0.037	0.037	0.037	0.037	0.037
**GSE20685**	<0.001	0.002	<0.001	<0.001	0.023	0.006	0.016	0.018	0.003
**GSE21653**	0.014	0.121	0.396	0.001	0.007	0.123	0.027	0.11	0.06
**GSE25055**	<0.001	<0.001	<0.001	<0.001	0.001	<0.001	<0.001	<0.001	<0.001
**GSE25065**	<0.001	<0.001	<0.001	<0.001	0.89	<0.001	0.597	<0.001	0.082
**c-index**									
**METABRIC D**	0.6182	0.6125	0.5969	0.6379	0.5961	0.6159	0.6015	0.5726	0.6279
**METABRIC V**	0.6004	0.5905	0.5860	0.6142	0.5724	0.5838	0.5679	0.5638	0.6069
**GSE2607**	**0.5661**	**0.6498**	**0.5700**	**0.6712**	0.5039	0.6342	0.5039	0.5039	0.5039
**GSE7390**	**0.5831**	0.5469	0.5795	0.5524	0.5604	0.5578	**0.5864**	0.4745	**0.5891**
**GSE11121**	**0.6126**	**0.6199**	0.5723	**0.6353**	**0.6631**	**0.6359**	**0.6429**	**0.6135**	0.586
**GSE17705**	**0.5845**	**0.569**	0.5232	0.5724	**0.6052**	**0.5865**	0.5112	0.5185	0.5543
**GSE20624**	0.5082	0.5063	0.5063	0.5989	0.5063	0.5063	0.5063	0.5063	0.5063
**GSE20685**	0.6064	0.5978	0.6213	0.6006	0.5762	0.5885	0.5798	0.5839	0.5989
**GSE21653**	**0.5701**	0.542	0.5209	**0.6121**	**0.5866**	0.5604	**0.5644**	0.568	0.5528
**GSE25055**	0.6384	0.6524	0.6301	0.6440	0.6022	0.6604	0.6126	0.6830	0.6472
**GSE25065**	**0.646**	**0.6567**	**0.653**	**0.6884**	0.5158	**0.6894**	0.5358	**0.667**	0.5766

Note: METABRIC D and METABRIC V are discovery and validation datasets from METABRIC study [91], and Other datasets represented by GSE ID are available from NCBI GEO database.

There are eight published signatures in the study including BRsig70 [3], BRsig76) [2], ONCO (Oncotype DX) [4], [5], TAMR13 [6], PAM50 [9], Genius [7], PIK3(PIK3CAGS278) [10], and GGI [8].

### Performance Measurements in BR1042

**Figure 2:**
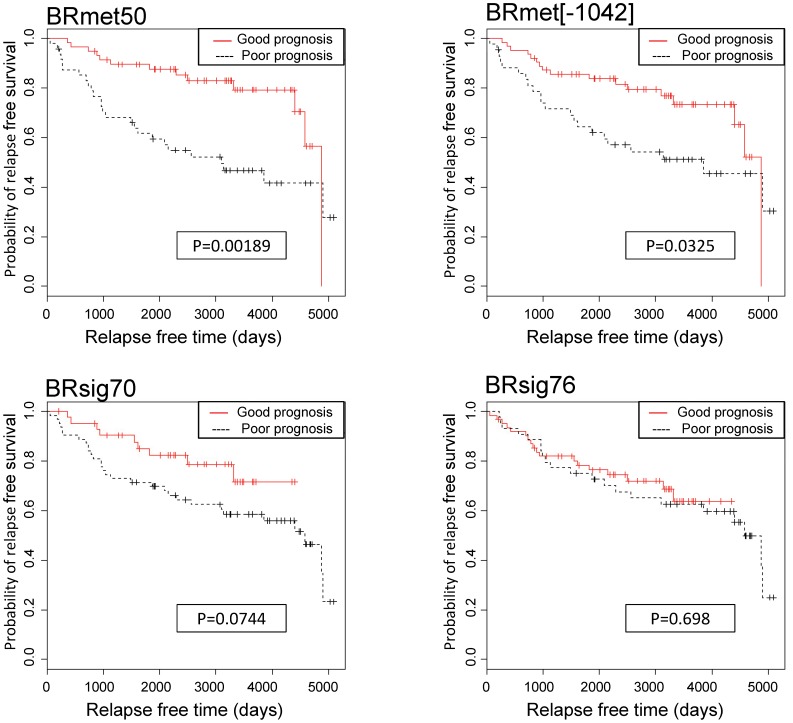
Kaplan-Meier analyses for relapse-free survival. Data from 108 tumors from the dataset BR1042 were stratified into two groups by BRsig70 and BRsig76 (bottom panels), the control signature (BRmet[-1042]) from the leave-one-out method, or BRmet50 (upper panels) gene expression profiles. In each survival plot, two types of relapse-free survival were compared: a poor prognosis group (black dashed line) and a good prognosis group (red solid line). The relapse-free time in days is displayed on the x-axis, and the y-axis shows the probability of relapse-free survival. The p*-*values indicate the statistical significance of survival time differences between the two groups.

Kaplan-Meier analysis was used to illustrate different relapse-free survival in BR1042 among the three types of signatures including BRmet50, one BRmet50 control signature, and two previously identified signatures (BRsig70 and BRsig76) (Figure 2). The results demonstrate a significant difference in relapse-free survival between the good and poor prognosis groups as predicted for the dataset BR1042 by BRmet50 as well as BRmet50 control signature (BRmet[-1042]) from the leave-one-out process (*p*<0.05). Among patients for whom BRmet50 predicted a good prognosis, the 10-year rate of relapse-free survival was 79% versus only 47% among those with a poor prognosis (Figure 2, upper left panel). The risk of relapse predicted by BRmet50 was significantly higher among patients in the poor prognosis group than that among those in the good prognosis group. However, for the same dataset, neither BRsig70 nor BRsig76 distinguished a significant difference in metastasis-free survival between the good and poor prognostic subgroups.

The performance of BRmet50 (c-index: 0.6573, p*-*value*:* 0.002) was better than those of BRsig70 and BRsig76 (c-index: 0.5839 or 0.5172, respectively, p-value >0.14) when examining the BR1042 dataset. Our results indicate that the predictive power of BRmet50 is robust and applicable across a wide range of independent datasets.

To assess whether BRmet50 association with prognosis outcome was specific, we generated 1,000 signatures of identical size (50 genes) using randomly selected genes from the human genome. All random signatures were tested in the same panel of 21 test datasets. After 1,000 random permutations of the gene signatures, the p-value distribution (-log p-value) from each test dataset was generated, and p-values from BRmet50 and the six other published cancer signatures were also plotted on the X-axis of the distribution plots (Figures S2 and S3).

Although some random signatures are significantly (*p*<0.05) associated with breast cancer outcomes in various datasets, the associations are stronger for the seven breast cancer signatures in more than half of the test datasets. These control results provide valid statistical support for their prognosis relevance. Furthermore, we noticed that most p-values from BRmet50 were on the far right side of the random p*-*value distributions (Figures S2 and S3). We then compared the patient outcome association of BRmet50 to those of 1,000 random signatures of identical size (Figure S2 and S3), and we confirmed that BRmet50 showed a stronger association than the vast majority of (>95%) random signatures. Thus, the probability of obtaining the same p-values as BRmet50 by chance in the same test datasets in Table 2 is significantly low (*p*<0.05).

### Predictive Power of BRmet50 Is Independent of Common Clinical and Pathological Covariates

Because dataset BR1141 [6] includes 269 patients with breast cancer and a full panel of common clinical and pathological covariates, we tested whether the association of BRmet50 with poor prognosis outcome was independent of established clinical and pathological criteria using the robust BR1141 dataset examined by Cox proportional-hazards models (Table 4 and Table S4). The association between BRmet50 and the risk of poor clinical outcome was significant regardless of tumor size, lymph-node status, or tamoxifen treatment (*p*<0.05). Furthermore, the BRmet50 could segregate tumors with intermediate differentiation or ER-positive into good and poor prognostic subcategories (hazard ratio for a poor prognosis: 2.5; *p*≤0.001) but not for those that were ER-negative. Neither BRsig70 nor BRsig76 was capable of stratifying tumors with either good or poor differentiation in any subset of BR1141 except tamoxifen treatment subset (Table 4). Because BR1141 was among the training datasets, we also tested a ‘leave-one-out’ BRmet50 control signature, and found identical significant associations (Table S4). The association between BRmet50 and relapse outcome in the BR1141 subset of patients without tamoxifen treatment is further described in the Methods S1.

**Table 4:** Hazard ratio risks for cancer relapse and log-rank tests in BR1141.

Clinicopathologic	BRmet50		BRsig70		BRsig76	
parameters	HR (95% CI)	HR P	HR (95% CI)	HR P	HR (95% CI)	HR P
**Tumor size**						
** T1**	2.6 (1.3–5.5)	0.009	1.5 (0.6–3.7)	0.386	1.0 (0.5–2.1)	0.942
** T2**	1.7 (1.0–2.8)	0.044	1.8 (0.9–3.8)	0.113	0.7 (0.4–1.2)	0.209
**Lymph-node involvement**						
** No**	2.3 (1.4–3.9)	0.001	1.6 (0.8–3.0)	0.193	0.8 (0.5–1.4)	0.511
** Yes**	2.0 (1.0–4.1)	0.053	2.8 (0.8–9.3)	0.089	0.6 (0.3–1.4)	0.245
**Tamoxifen treatment**						
** No**	2.6 (1.4–5.0)	0.004	2.1 (1.0–4.6)	0.063	1.1 (0.5–2.0)	0.869
** Yes**	2.2 (1.2–3.9)	0.007	1.7 (0.7–4.0)	0.230	0.6 (0.3–1.0)	0.041
**Differentiation**						
** Good**	2.3 (0.6–8.4)	0.196	2.4 (0.8–7.2)	0.121	1.3 (0.4–3.8)	0.682
** Intermediate**	2.5 (1.5–4.3)	0.001	1.6 (0.8–3.4)	0.219	0.7 (0.4–1.2)	0.194
** Poor**	1.4 (0.6–3.4)	0.442	0.2 (0–1.8)	0.172	0.5 (0.2–1.1)	0.086
**ER status**						
** Negative**	1.4 (0.5–4.0)	0.495	2.2(0.3–16.3)	0.456	0.9 (0.3–2.3)	0.782
** Positive**	2.5 (1.6–4.0)	<0.001	1.8 (1.0–3.3)	0.050	0.7 (0.4–1.1)	0.103

The 269 patients with breast cancer included in the BR1141 dataset were stratified according to tumor size, lymph-node status, tamoxifen treatment, histological grade, and ER status. A univariate Cox proportional-hazards model was used to evaluate the association of individual signatures (i.e., the BRmet50, BRsig70, or BRsig76) with the clinical outcome in each category.

T1 denotes a tumor with size less than or equal to 2.0 cm, and T2 denotes a tumor with size larger than 2.0 cm. HR (95% CI): hazard ratio value (95% confidence interval). HR P: hazard ratio p-value.

Five of the 21 datasets used for evaluating BRmet50 performance (BR1042, BR1095, BR1128, BR1141, GSE7390) represented 1,183 tumors and had data on a common set of clinicopathologic characteristics including tumor size, grade, lymph node status, and Nottingham Prognostic Index (NPI) [32], [33]. Univariate and multivariate analyses of these five validation sets were performed to further evaluate the performance of BRmet50 compared with other prognostic factors, namely, BRsig70, BRsig76, age, tumor size, grade, lymph node status, and NPI. The unadjusted (Table S5) and adjusted (Table 5 and Table S6) hazard ratios of these factors and signatures were determined.

**Table 5:** Multivariate analysis of disease risk among patients with breast cancer.

	BRmet50		BRsig70		BRsig76	
Datasets	HR (95% CI)	HR P	HR (95% CI)	HR P	HR (95% CI)	HR P
**BR1042**	3.1 (1.4–7.0)	<0.01	1.7 (0.7–3.9)	0.23	0.8 (0.4–1.7)	0.54
**BR1095**	1.8 (1.1–2.9)	0.02	1.5 (0.9–2.5)	0.16	1.3 (0.8–2.2)	0.26
**BR1128**	2.0 (1.0–3.9)	0.03	1.4 (0.8–2.7)	0.27	1.2 (0.6–2.3)	0.49
**BR1141**	2.3 (1.4–3.6)	<0.01	1.6 (0.9–2.9)	0.13	0.6 (0.4–1.0)	0.05
**GSE7390**	2.5 (1.4–5.0)	<0.01	1.1 (0.6–2.0)	0.76	2.0 (1.1–3.3)	0.03

The HR and p-values for each signature were adjusted by age, grade, tumor size, LN, ER, and NPI.

Age and tumor diameter were modeled as continuous variables; the hazard ratio is for each increase of 1 cm. in diameter or for each 1-year increase in age. HR: hazard ratio with 95% confidence interval; HR P: hazard ratio p-value.

Univariate Cox proportional-hazards analysis demonstrated that BRsig70, BRsig76, or any individual common prognostic factor (tumor size, grade, lymph node status, or NPI) could not successfully predict cancer prognoses in all five datasets. However, BRmet50 was uniquely able to significantly differentiate tumor samples into two prognostic groups in all five validation sets. The prognostic value of BRmet50 was greater than each of the established risk factors (Table S5). For example, optimal unadjusted hazard ratios (HR) (high risk vs. low risk) in BR1128 were 2.8 (95% CI: 1.5–4.9; p<0.001) (BRmet50 control), 1.9 (95% CI: 1.1–3.3; p = 0.01) (BRmet70), 2.0 (95% CI: 1.1–3.5; p = 0.02) (BRmet76), and 2.2 (95% CI: 1.6–2.9; p<0.01) (NPI), respectively. The data suggested that the BRmet50 was more efficient at predicting relapse-free survival in BR1042, BR1141, and GSE7390 and disease-free survival in BR1095 and BR1128 than established prognostic factors.

Multivariate Cox proportional-hazards analysis was used to determine if BRmet50, BRsig70, or BRsig76 added independent prognostic information to other standard clinicopathological features. In this multivariate Cox proportional-hazards analysis (Table 5), significant associations (*p*<0.05) were observed in all five test datasets between BRmet50 and patient relapse-free or disease-free time after adjustment for standard clinical covariates. Thus, BRmet50 contributed new and important prognostic information beyond that provided by established clinical predictors. For the most part, BRsig70 and BRsig76 showed no significant associations in these analyses.

### Predictive Power of BRmet50 in Other Cancer Types

Because BRmet50 successfully predicted breast cancer prognosis and because some molecular oncogenic events are conserved among multiple cancer types [34], we hypothesized that BRmet50 may represent a conserved transcriptional profile for poor prognosis in multiple cancer types.

To examine the prognostic specificity of BRmet50, we investigated whether BRmet50 could predict prognosis in other epithelial cancers such as colon, lung, or prostate cancer. Three datasets, one for each cancer type: colon cancer (n = 73) [35], lung cancer (n = 441) [36], and prostate cancer patients (n = 596) (Table 6) [37] were subjected to univariate and multivariate analyses. On the basis of gene expression signatures (BRsig70, BRsig76, or BRmet50), 1,110 patient samples were segregated into two groups (Table 6). All three signatures failed to predict cancer relapse in colon cancer [35] (p>0.05). However, BRmet50 but neither BRsig70 nor BRsig76 successfully predicted disease specific survival in prostate cancer and relapse-free survival in lung cancer (*p*<0.01), suggesting that transcriptional profiles for poor-prognosis may be more conserved in breast, lung, and prostate cancer. In the lung cancer dataset, the good prognosis groups predicted by BRmet50 had the highest relapse-free survival (>40% and *p*<0.01) among the 3 signatures. We also determined whether the association between the three signatures and the clinical outcomes in patients with prostate, lung, and colon cancer was independent of established clinical and pathological criteria (Table 6). The results suggest that BRmet50 might serve as a prognostic biomarker for both breast and non-breast cancer and may represent a conserved transcriptional profile among multiple cancer types.

**Table 6:** Univariate and multivariate analysis in lung, prostate, and colon cancer.

		BRmet50		BRsig70		BRsig76	
**Cancer type**	**Analysis**	**HR (95% CI)**	**HR P**	**HR (95% CI)**	**HR P**	**HR(95% CI)**	**HR P**
**Lung** [36]	**Univariate**	1.7 (1.3–2.4)	**<0.01**	1.2 (0.8–1.9)	0.37	1.2 (0.8–1.9)	0.37
	**Multivariate** *	1.8 (1.2–2.5)	**<0.01**	1.2 (0.7–1.9)	0.51	1.2 (0.7–1.9)	0.51
**Prostate** [37]	**Univariate**	0.4 (0.3–0.6)	**<0.01**	1.2 (0.8–1.8)	0.34	0.7 (0.5–1.0)	0.07
	**Multivariate** **	0.6 (0.3–1.0)	**0.04**	1.3 (0.8–2.1)	0.27	0.9 (0.5–1.4)	0.52
**Colon** [35]	**Univariate**	1.3 (0.4–4.7)	**0.66**	0.5 (0.1–1.9)	0.32	0.5 (0.2–1.9)	0.32
	**Multivariate** ***	1.4 (0.4–5.0)	**0.62**	0.5 (0.1–1.8)	0.31	0.5 (0.1–1.8)	0.30

* Adjusted factors in lung cancer: age, gender, chemotherapy treatment, radiation treatment, smoking habits, and tumor stage.

** Adjusted factors in prostate cancer: age, tumor stage, ploidy, and PSA relapse.

*** Adjusted factor in colon cancer: age.

## Discussion

Data generated by high-throughput transcriptional studies of cancer has rapidly accumulated and there is increasing interest in translating this information into clinical value. Although single-study analysis can be informative, it is often affected by inherent limitations. These limitations can be overcome by combining related independent studies into a meta-analysis. Our study demonstrated that heterogeneous signatures from individual cancer studies can be systematically organized into a meta-signature (BRmet50) based on their intrinsic data similarities by a novel meta-analysis strategy (iterative EXALT). This meta-analysis approach can increase statistical power, minimize false discovery, reduce batch effects, and improve the generalizability of the findings. The value of the BRmet50 signature was evaluated in terms of predicting prognoses in breast and other cancers.

There are two strategies for meta-analysis of transcriptional datasets: *data combination* and *data integration* methods. The *data combination* method is a comprehensive reanalysis of the primary data by merging data from multiple studies [38]–[46]. This method is powerful because all of the information in the datasets is used. However, this power comes with some risks such as the necessity to model heterogeneity between datasets. Specifically, use of this approach often requires an ad-hoc normalization of the raw data files [47], [48] followed by explicitly modeling the inter-study variability [45], [49]. The *data integration* method compares gene lists from any expression platform filtered according to p-values or rank combination [50], [51]. The large capacity is not dependent upon the methods used for the initial data processing [52]; heterogeneous datasets become comparable after simplification of raw gene expression values to gene lists, but it comes with the risk that a significant amount of information might be lost. This method has been successfully implemented in a variety of analysis tools such as Venn diagrams, L2L [53], LOLA [54], GeneSigDB [55], Oncomine [56], Connectivity Map (CMAP) [57], and our own novel method called EXALT [21].

Iterative EXALT helped us understand the relationship between the intrinsic signature data similarities and signature-associated phenotypes. When the clustered signature phenotypes in Table 1 were cross-checked with all source phenotypes in Table S1, it was confirmed that the datasets with the same sample phenotypes were not necessary to generate signatures with significant data similarity. All data integration methods except EXALT have a shared challenge in how to collect suitable profiling datasets from heterogeneous gene expression studies. These methods typically analyze a limited number of data sets brought together through a prior knowledge-based search (inclusion/exclusion criteria) rather than by intrinsic data similarities [20]. Even though such approaches can ensure that the patient populations or sample phenotypes are similar or homogeneous, they are inadequate given that (1) they can miss valuable datasets and (2) they can include incorrect data sets having no data similarity, resulting in abnormal heterogeneous expression profiles. This characteristic can negatively affect the profile performance, robustness, and applicability. To collect homogeneous datasets for any meta-analysis, it is still a big hurdle when lacking a data-driven quantitative evaluation for inclusion/exclusion criteria [19]. To solve this problem and exploit the enormous wealth of available data to their full potential, iterative EXALT can systematically integrate available transcriptional datasets in public domains (Table S1) based on intrinsic data similarities. The unique processes performed by the iterative EXALT method include gathering homogeneous signatures for meta-analysis (Table 1), consolidating homogeneous signatures, and discovering reliable and recurrent meta-signatures for given diseases or biologically related phenotypes (Figure 1). These important features are not present in our previous EXALT program [21], [22], nor can they be found in any other data integration methods.

The power of the iterative EXALT is illustrated in the identification of a meta-signature (BRmet50). In our meta-validation of BRmet50, we found that distinct gene expression signatures have a common significant predictive value in more than half of the breast cancer studies (Table 2, Table 3, and Figure S2 and S3). This agreement supports the notion that the limited overlap in gene identity among gene expression profiles does not affect similar prognostic performance in breast cancer [58]. However, unlike other studies in which only a few test datasets were examined [59], [60], our current study included a large number of test datasets. We found that some well-established cancer signatures were not significant predictors in several published breast cancer survival studies (Table 2 and Table 3). Further, when adjusted for major prognostic clinical covariates, neither BRsig70 nor BRsig76 was able to discriminate between good and poor prognosis groups in multiple breast cancer datasets (Table 5). This observation agrees with the notion that BRsig70 is a predictor of early relapse and is of limited clinical utility in breast cancers [11], [15], [61]–[66]. One explanation is that BRsig70 and BRsig76 had been previously validated only in a few datasets with the selected patient subsets (e.g., patients with lymph-node-negative status) [11], [61], [67]. A large prospective clinical trial (MINDACT) is now being carried out [68] to test whether BRsig70 can predict prognosis in patients with node-negative as well as those patients with one to three positive lymph node to avoid chemotherapy [15]. Our results emphasize the need to perform additional validation studies of transcriptional biomarkers, including a demonstration of their value beyond common histopathological predictors [5], [69], for extrapolation to a more general patient population [70]–[74]. A meta-analysis strategy combining both discovery and validation of transcriptional biomarkers may be well-suited to accomplish these goals.

A previous report [59] suggested that a large percentage (>50%) of random gene expression signatures were significantly associated with breast cancer outcome in two breast cancer datasets (designated here as BR2411 [11] and BR1141 [6]). We generated 1,000 random signatures with identical in size to BRmet50 from the human genome and examined them using 21 validation datasets (Figures S2 and S3). Based on the random p-value distributions, we found that the distributions were heterogeneous. Some datasets such as BR18347175 and GSE20624 had unusual skewed distributions of random p-values and a high percentage (50% or higher) of random signatures that were significantly associated with breast cancer outcome at *p*<0.05. However, for the majority of the other validation datasets, outcome association of BRmet50 and most published cancer signatures showed stronger associations than the median of random signatures. On average, the association of BRmet50 with disease outcome was stronger than that of the top 5% random signatures (Figures S2 and S3).

One important observation from this large scale validation of results is that a random signature may produce significant outcome associations (*p*<0.05) in a small number of test datasets, but it is still very difficult for a random signature to repeatedly yield significant results by chance in a majority of 21 test datasets. Out of 1,000 random signatures, there were only 13 (1.3% of random signatures) that generated significant predictions (*p*<0.05) from more than 10 out of 21-test datasets (>50%). However, for the same 21 validation datasets, 100% of the tests of BRmet50 and more than 50% of the tests of the six other known cancer signatures were significant. Abiding to this criterion, the probability that a random signature achieves the similar level of performance as the BRmet50 by chance is low (*p*<0.013). Clearly, our study emphasizes the importance of large-scale validation tests.

A 21-gene signature (Oncotype DX) is a diagnostic test that quantifies the likelihood of relapse of tamoxifen-treated, lymph node-negative breast cancer using a recurrence score method [4]. The recurrence score is derived from the RT-PCR based reference-normalized expression measurements for 16 cancer-related genes. The panel of 21 genes in Oncotype DX includes some well-known biomarker genes for breast cancer subtypes and prognosis prediction such as Ki67, HER2, ER, and PGR. This has raised concern about whether it truly adds independent prognostic information beyond other standard clinicopathological covariates [5], [69]. We did not apply the Oncotype recurrence score formula directly to gene expression values described in this study. In order to make comparisons between BRmet50 and this widely used prognostic marker, we examined the prognosis prediction values of Oncotype DX signature and the other six well-known cancer signatures in all 21 test datasets (Figures S2 and S3 and Table 3) using random signature simulations as negative controls. The results suggest that Oncotype DX is a strong predictor with significant predictions in 80% of test datasets.

Because breast cancer is such a heterogeneous disease, most recent studies have taken the molecular heterogeneity of breast cancer into account in their predictions [7], [75]. As a general prognosis predictor in cancer, BRmet50 is a meta-signature derived from datasets representing heterogeneous cancer subtypes, and BRmet50 therefore represents mixed gene expression profiles of various breast cancer subtypes. During the univariate analysis of breast cancer subtypes (Table 4), we noticed that BRmet50 could be used to segregate ER-positive (luminal tumors) and intermediate grade tumors regardless of tumor size and lymph-node status into good and poor prognostic subcategories (hazard ratio for a poor prognosis: 2.5; *p*<0.001) but not for those with ER-negative status or those with high-grade (Table 4). These prognostic features are consistent with those of many other breast cancer prognostic signatures [60], [76]–[80] but different than those of subtype-specific prognostic predictors (GENIUS) that can be applied to breast cancer samples with ER-negative or HER2-negative status [7]. When BRmet50 was used in the subtype classification model [75] we found that BRmet50 could inform subtype classification but its prediction strength was not as robust as the three-gene model [75].

In summary, we have developed and demonstrated the utility of a novel data similarity-based meta-analysis strategy for deducing a transcriptional meta-signature with enhanced prognostic value in breast cancer. We report a novel meta-signature, BRmet50, which has a superior capability to predict clinical outcomes in 21 breast cancer transcriptional profiling datasets. Furthermore, BRmet50 can distinguish prognostic subsets of patients with ER-positive breast cancer or intermediate-grade breast cancer regardless of lymph-node and tamoxifen treatment status. Finally, we demonstrated that BRmet50 has predictive value in other cancer types (prostate and lung), suggesting that different cancers may share common transcriptional elements that influence their clinical behaviors. Additional prospective studies will be valuable in determining the clinical value of BRmet50 in breast cancer patient subsets and other cancers.

## Methods

The methods used for signature extraction, developing the signature databases, and EXALT analysis were previously reported [21], [22]. Iterative EXALT analysis for clustering and assembling signatures is described in the result section (Figure 1) and the Methods S1.

### Patient Data

Patient information, both clinical data and gene expression data for signature identification and validation, were obtained from independently published human cancer studies and the Gene Expression Omnibus (GEO) provided by the National Center for Biotechnology Information (NCBI) [81] as described in Table S1, Table 1, Table 2, and Table 6. The meta-signature (BRmet50) was derived from meta-analysis of breast cancer gene expression profiles from 223 breast cancer training datasets (Table S1). Leave-one-out cross validation was used to prepare BRmet50 control signatures from nine training datasets (Table S2) as described in the Methods S1. To provide an evaluation of the iterative EXALT approach and meta-signature, 21 datasets (Table 2) containing 6,011 breast tumor samples were retrospectively examined by survival analyses [2], [3], [6], [8], [11], [25], [27], [28], [82]–[91]. Of them, 10 are from 223 training datasets (Table 1 and Table 2), and the other 11 (“validation datasets” in Table 2) are independent validation datasets not included in the 223 training datasets (Table S1).

To ensure quality in the test survival data sets derived from published breast cancer studies, we applied the “rule of fifty” [92]–[94] as an inclusion criteria. Specifically, an included dataset must have at least 50 samples with survival data (designated as survival samples) and a minimum of 10 events. To ensure a valid sample size for a survival analysis, at least 60% of the samples were required to have survival information. Thus, missing data (censored survival data) was controlled to a minimal level. The average follow-up length was 14 years across 21 datasets.

### Statistical Analysis

Our statistical approach, as illustrated in Figure S1, assessed the ability of the identified meta-signature BRmet50 to serve as a survival time predictor. First, hierarchical clustering of the BRmet50 gene profiles in each test dataset was performed and visualized using the open-source desktop program (version 1.5.0.Gbeta) developed at Vanderbilt University. Spearman rank correlation was used to measure the similarities in gene expression profiles among patient samples. Unsupervised hierarchical clustering based on average linkage was performed to group the patient samples. The group assignments for the patient samples were determined in each dataset based on the first bifurcation of the clustering sample dendrogram [30]. Using disease outcomes, Kaplan-Meier curves for the two groups were compared. Log-rank tests and c-index measurements were conducted for the two groups’ survival difference. The Cox proportional hazards model was applied to each dataset for both univariate and multivariate survival analyses. All these analyses were carried out with the open-source R software, version 2.14.1 (www.r-project.org).

For general prognosis performance evaluation of various signatures in full datasets and subsets, p-values from log-rank tests and from univariate and multivariate Cox proportional hazard models were evaluated. Various disease outcomes (e.g., relapse, distant metastasis, or death) were used as clinical end points (Table 2). The estimated hazard ratio (HR), its 95% confidence interval (CI), and the p-value allowed us to directly compare the performances of different signatures (Figure S1). For graphical representation, Kaplan-Meier curves of survival probability were plotted for each subgroup.

## Supporting Information

Figure S1 **Flow chart of statistical methods for validation.** Four types of signatures were used in this study: (1) BRmet50; (2) BRmet50 control signatures from “Leave-one-out” process; (3) BRsig70, BRsig76 and other six known signatures for breast cancer prognosis; and (4) 1,000 random signatures of identical in size to BRmet50. Gene expression signatures were used for unsupervised hierarchical clustering. Sample group assignments were determined in each data set based on the sample clustering dendrogram. Gene expression-based sample groups together with patient survival data and clinicopathological variables in cancer were used to determine the signature prognostic performance in survival analyses. The survival analyses include log-rank tests and Cox proportional hazards regression models (univariate and multivariate models). All signatures were validated in 21 breast cancer (BR) data sets (Table 2, Figures S2, S3, and 2 and Table 3), and one breast cancer data set (BR1141) was further analyzed among breast cancer subsets (Table 4 and Table S4). BRmet50, BRsig70, and BRsig76 were examined to determine whether they were independent of common clinicopathologic factors in breast cancer (Table 5, Tables S5, S6) and three other three cancer types (Table 6).
(TIFF)

Figure S2 **Comparison of cancer signatures and random signatures (part 1).** 12 datasets were tested individually with 1,000 random signatures and seven known cancer signatures. Each panel is labeled with its respective test dataset ID and depicts the distribution of *p*-values from1,000 random signatures identical in size to BRmet50 (50 genes). The x-axis denotes the reciprocal logarithm of *p*-value (-log [*p*-value]) from survival analyses. Colored arrowheads represent the seven known cancer signatures and point to the *p*-value locations in the random *p*-value distributions.
(TIFF)

Figure S3 **Comparison of cancer signatures and random signatures (part 2).** Nine datasets were tested individually with 1,000 random signatures and seven known cancer signatures. Each panel is labeled with its respective test dataset ID and depicts the distribution of *p*-values from1,000 random signatures identical in size to BRmet50 (50 genes). The x-axis denotes the reciprocal logarithm of *p*-value (-log [*p*-value]) from survival analyses. Colored arrowheads represent the seven known cancer signatures and point to the *p*-value locations in the random *p*-value distributions.
(TIFF)

Table S1 **Breast cancer dataset source and derived signature phenotypes.**
(DOCX)

Table S2 **List of breast cancer meta-signatures.**
(DOCX)

Table S3 **Annotation of genes in BRmet50.**
(DOCX)

Table S4 **Hazard ratio risks and log-rank tests in BR1141.**
(DOCX)

Table S5 **Comparison of signatures with common clinicopathologic factors by univariate hazard ratio model.**
(DOCX)

Table S6 **Multivariate analysis of relapse risk among patients with breast cancer.**
(DOCX)

Methods S1 **Supplemental methods.**
(DOC)
