# Supplementary figures and images for: A Data Similarity-Based Strategy for Meta-analysis of Transcriptional Profiles in Cancer
Source: PLoS One. 2013 Jan 29;8(1):e54979. doi: 10.1371/journal.pone.0054979 (PMC3558433; doi:10.1371/journal.pone.0054979)

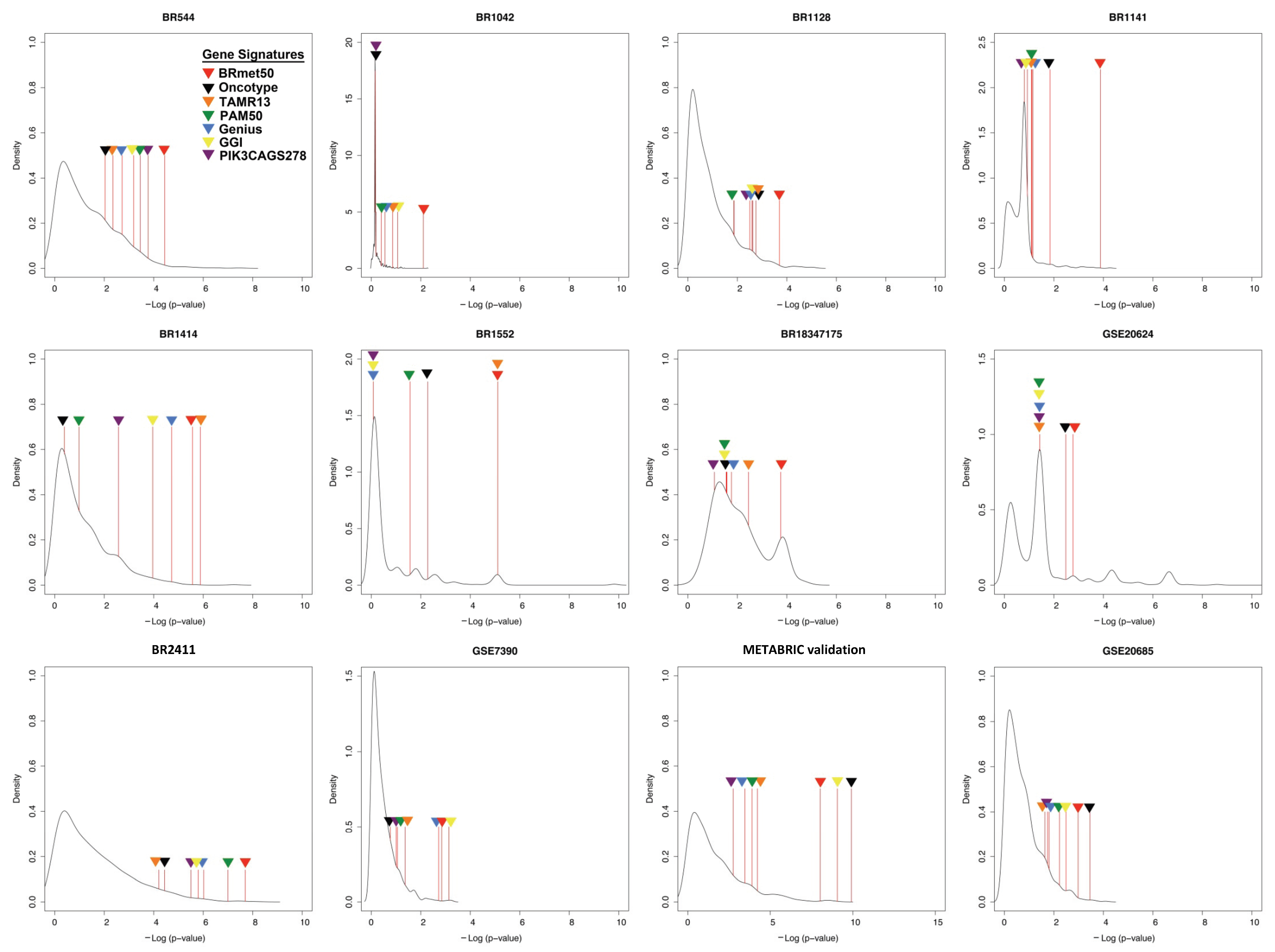

Supplement: Figure S2 — Comparison of cancer signatures and random signatures (part 1). 12 datasets were tested individually with 1,000 random signatures and seven known cancer signatures. Each panel is labeled with its respective test dataset ID and depicts the distribution of p-values from1,000 random signatures identical in size to BRmet50 (50 genes). The x-axis denotes the reciprocal logarithm of p-value (-log [p-value]) from survival analyses. Colored arrowheads represent the seven known cancer signatures and point to the p-value locations in the random p-value distributions. (TIFF) [file pone.0054979.s002.tiff]

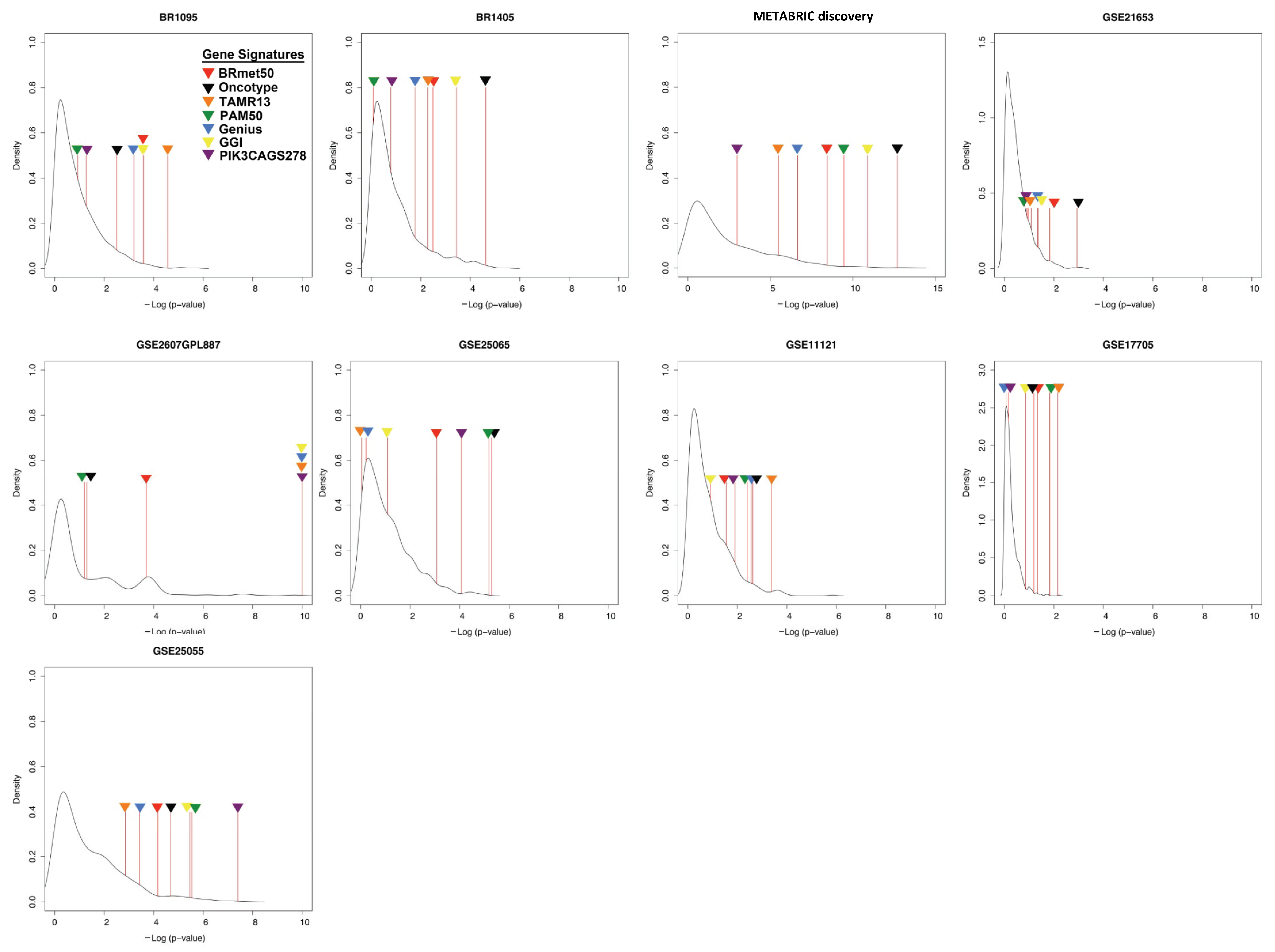

Supplement: Figure S3 — Comparison of cancer signatures and random signatures (part 2). Nine datasets were tested individually with 1,000 random signatures and seven known cancer signatures. Each panel is labeled with its respective test dataset ID and depicts the distribution of p-values from1,000 random signatures identical in size to BRmet50 (50 genes). The x-axis denotes the reciprocal logarithm of p-value (-log [p-value]) from survival analyses. Colored arrowheads represent the seven known cancer signatures and point to the p-value locations in the random p-value distributions. (TIFF) [file pone.0054979.s003.tiff]
