# Supplementary material for: A Data Similarity-Based Strategy for Meta-analysis of Transcriptional Profiles in Cancer
Source: PLoS One. 2013 Jan 29;8(1):e54979. doi: 10.1371/journal.pone.0054979 (PMC3558433; doi:10.1371/journal.pone.0054979)
Supplement: Table S1 — Breast cancer dataset source and derived signature phenotypes. (DOCX) [file pone.0054979.s004.docx]

**Table S1. Breast cancer dataset source and derived signature phenotypes**

| **Study Name** | **PMID** | **Signature Phenotypes** |
| --- | --- | --- |
| Adler AS et al Nat Genet 2006 | 16518402 | CSN5, GFP, MYC, and MYC+CSN5 |
| Anders CK et al PLoS ONE 2008 | 18167534 | age comparison ranging from 45 to 65 years old |
| Anders CK et al PLoS ONE 2008 | 18167534 | ductal NOS and infiltrating duct lobular carcinoma |
| Anders CK et al PLoS ONE 2008 | 18167534 | ER negative vs. ER positive |
| Anders CK et al PLoS ONE 2008 | 18167534 | mono- , non- , and tri- chemotherapy |
| Anders CK et al PLoS ONE 2008 | 18167534 | node negative vs. node positive |
| Anders CK et al PLoS ONE 2008 | 18167534 | nuclear grade 1, 2, and 3 |
| Anders CK et al PLoS ONE 2008 | 18167534 | pathological stages 1, 2A, 2B, and 3B |
| Anders CK et al PLoS ONE 2008 | 18167534 | PR negative vs. PR positive |
| Anders CK et al PLoS ONE 2008 | 18167534 | recurrence vs. no recurrence |
| Anders CK et al PLoS ONE 2008 | 18167534 | TNM stages 1A, 1B, 1C, and 2 |
| Anders CK et al PLoS ONE 2008 | 18167534 | tumor size comparison between 2.0cm to 5.0 cm |
| Anders CK et al PLoS ONE 2008 | 18167534 | undifferentiated, moderately, and poorly differentiated |
| Anders CK et al PLoS ONE 2008 | 18167534 | without hormone therapy vs. with hormone therapy |
| ang E et al Lancet 2003 | 12747878 | lymph node status in breast cancer |
| ang E et al Lancet 2003 | 12747878 | relapse status in breast cancer |
| ardson AL et al Cancer Cell 2006 | 16473279 | BRCA mutation negative vs. BRCA mutation positive |
| ardson AL et al Cancer Cell 2006 | 16473279 | ER negative vs. ER positive |
| ardson AL et al Cancer Cell 2006 | 16473279 | HER2 negative vs. HER2 positive |
| ardson AL et al Cancer Cell 2006 | 16473279 | PR negative vs. PR positive |
| Bhati R et al Am J Pathol 2008 | 18403594 | vascular cells from breast tumor samples |
| Bild AH et al Nature 2006 | 16273092 | Bcat1-9, E2F3 1-10, RAS1-10, GFP1-10, Myc1-10, and Src1-7 |
| Bild AH et al Nature 2006 | 16273092 | ER status levels 0 1 vs. ER status level 2 3 |
| Bild AH et al Nature 2006 | 16273092 | ER status level 0 vs. ER status levels 1 2 3 |
| Bild AH et al Nature 2006 | 16273092 | squamous and adenocarcinoma |
| Chanrion M et al Clin Cancer Res 2008 | 18347175 | ER negative vs. ER positive |
| Chanrion M et al Clin Cancer Res 2008 | 18347175 | grades 1, 2, and 3 |
| Chanrion M et al Clin Cancer Res 2008 | 18347175 | no distant metastasis vs. distant metastasis |
| Chanrion M et al Clin Cancer Res 2008 | 18347175 | no local recurrence vs. local recurrence |
| Chanrion M et al Clin Cancer Res 2008 | 18347175 | node negative vs. node positive |
| Chanrion M et al Clin Cancer Res 2008 | 18347175 | PR negative vs. PR positive |
| Chanrion M et al Clin Cancer Res 2008 | 18347175 | x-ray and Tam, Tam, x-ray Tam and LHR |
| Chapman SC et al BMC Dev Biol 2007 | 17663788 | node status and subtypes |
| Chi JT et al PLoS Genet 2007 | 17907811 | smooth muscle tissue set 1 and breast cancer prognosis |
| Chi JT et al PLoS Genet 2007 | 17907811 | smooth muscle tissue set 2 and breast cancer porgnosis |
| Chi JT et al PLoS Genet 2007 | 17907811 | smooth muscle tissue set 3 and breast cancer prognosis |
| Cicatiello L et al J Mol Endocrinol 2004 | 15171711 | estrogen actions in breast cancer cells from 1 h to 32h |
| Cicatiello L et al J Mol Endocrinol 2004 | 15171711 | sham-treated p53 RNAi and Dox-treated cell lines |
| Dairkee SH et al BMC Genomics 2004 | 15260889 | mammary epithelial tumor tissue and breast cancer cell lines |
| Dittmer A et al J Biol Chem 2006 | 16551631 | cells, transfected with siPTHrP vs. control siRNA |
| er WR et al Pharmacogent Genomics 2007 | 17885619 | breast tumor with letrozole treatmentl for 10 to 14 days |
| Farmer P et al Oncogene 2005 | 15897907 | AR negative vs. AR positive |
| Farmer P et al Oncogene 2005 | 15897907 | ER negative vs. ER positive |
| Finak G et al Breast Cancer Res 2006 | 17054791 | ER status |
| Finak G et al Breast Cancer Res 2006 | 17054791 | grades 2 and 3 |
| Finak G et al Breast Cancer Res 2006 | 17054791 | HER2 status |
| Finak G et al Breast Cancer Res 2006 | 17054791 | node status |
| Finak G et al Breast Cancer Res 2006 | 17054791 | normal epithelium and stroma adjacent to I.D.C. |
| Finak G et al Breast Cancer Res 2006 | 17054791 | post-, pre- , and surgical menopause |
| Finak G et al Nat Med 2008 | 18438415 | ER positive vs. ER negative |
| Finak G et al Nat Med 2008 | 18438415 | ERBB2 positive vs. ERBB2 negative |
| Finak G et al Nat Med 2008 | 18438415 | grade 1 vs. grade 2 |
| Finak G et al Nat Med 2008 | 18438415 | node positive vs. Node negative |
| Finak G et al Nat Med 2008 | 18438415 | PR positive vs. PR negative |
| Finak G et al Nat Med 2008 | 18438415 | tumor stroma vs. matched normal stroma |
| Foekens JA et al J Clin Oncol 2006 | 16505412 | highly, morderately, and poorly differentiated tumors |
| Foekens JA et al J Clin Oncol 2006 | 16505412 | with recurrence vs. without recurrence |
| Gruvberger et al Clin Cancer Res 2007 | 17404078 | ER alpha negative vs. ER alpha positive |
| Hegde PS et al Mol Cancer Ther 2007 | 17513611 | Latinib treatment in time series |
| Hernández-V H et al Int J Cancer 2006 | 16557594 | MCF7 treated by 5-FU in time series |
| Herschkowitz JI et al Breast Cancer Res 2008 using GPL1390 | 18782450 | breast Cancer subtypes |
| Herschkowitz JI et al Breast Cancer Res 2008 using GPL1390 | 18782450 | grades 1, 2, and 3 |
| Herschkowitz JI et al Breast Cancer Res 2008 using GPL1390 | 18782450 | less than 45, 45 to 65, more than 65 |
| Herschkowitz JI et al Breast Cancer Res 2008 using GPL1390 | 18782450 | lymph node status |
| Herschkowitz JI et al Breast Cancer Res 2008 using GPL1390 | 18782450 | no relapse vs. relapse or die of disease-related death |
| Herschkowitz JI et al Breast Cancer Res 2008 using GPL1390 | 18782450 | tumor size comparison and local invasion |
| Herschkowitz JI et al Breast Cancer Res 2008 using GPL885 | 18782450 | breast cancer subtypes |
| Herschkowitz JI et al Breast Cancer Res 2008 using GPL885 | 18782450 | metastasis vs. no metastasis |
| Herschkowitz JI et al Breast Cancer Res 2008 using GPL887 | 18782450 | breast cancer subtypes |
| Herschkowitz JI et al Breast Cancer Res 2008 using GPL887 | 18782450 | ER negative vs. ER positive |
| Herschkowitz JI et al Breast Cancer Res 2008 using GPL887 | 18782450 | grade 1, 2, and 3 |
| Herschkowitz JI et al Breast Cancer Res 2008 using GPL887 | 18782450 | no relapse vs. relapse or die of disease |
| Herschkowitz JI et al Breast Cancer Res 2008 using GPL887 | 18782450 | tumor sizes and direct extension to chest wall or skin |
| Herschkowitz JI et al Breast Cancer Res 2008 using GPL1390 | 18782450 | ER negative vs. ER positive |
| Herschkowitz JI et al Breast Cancer Res 2008 using GPL885 | 18782450 | 45 to 65 vs. older than 65 |
| Herschkowitz JI et al Breast Cancer Res 2008 using GPL885 | 18782450 | ER negative vs. ER positive |
| Herschkowitz JI et al Breast Cancer Res 2008 using GPL887 | 18782450 | younger than 45 vs. 45 to 65 |
| Hoadley KA et al BMC Genomics 2007 | 17663798 | relapse and breast cancer subtypes |
| Hoadley KA et al BMC Genomics 2007 | 17663798 | Control and treatment in time series |
| Hoadley KA et al BMC Genomics 2007 | 17663798 | ER status and breast cancer subtypes |
| Hoadley KA et al BMC Genomics 2007 | 17663798 | grade status and breast cancer subtypes |
| Hu Z et al BMC Genomics 2006 using GPL1390 | 16643655 | ER negative vs. ER positive |
| Hu Z et al BMC Genomics 2006 using GPL1390 | 16643655 | node negative vs. node positive |
| Hu Z et al BMC Genomics 2006 using GPL887 | 16643655 | ER negative vs. ER positive |
| Hu Z et al BMC Genomics 2006 using GPL887 | 16643655 | node negative vs. node positive |
| Hu Z et al BMC Genomics 2006 using GPL1390 | 16643655 | breast cancer subtypes |
| Hu Z et al BMC Genomics 2006 using GPL1390 | 16643655 | grades 1, 2, and 3 |
| Hu Z et al BMC Genomics 2006 using GPL1390 | 16643655 | no relapse vs. relapse |
| Hu Z et al BMC Genomics 2006 using GPL885 | 16643655 | ER negative and breast cancer subtypes |
| Hu Z et al BMC Genomics 2006 using GPL887 | 16643655 | ER status and breast cancer subtypes |
| Hu Z et al BMC Genomics 2006 using GPL887 | 16643655 | grades 1, 2, and 3 |
| Hu Z et al BMC Genomics 2006 using GPL887 | 16643655 | no relapse vs. relapse |
| ider J et al Int J Cancer 2006 | 17019712 | ER positive vs. ER negative |
| ider J et al Int J Cancer 2006 | 17019712 | PR positive vs. PR negative |
| Ivshina AV et al Cancer Res 2006 using GPL96 | 17079448 | luminal A, luminal B, Basal, ERBB2, and normal like |
| Ivshina AV et al Cancer Res 2006 using GPL96 | 17079448 | 1-like, 2a-, 2b-, and 3-like |
| Ivshina AV et al Cancer Res 2006 using GPL96 | 17079448 | ELSTON grades 1, 2, and 3 |
| Ivshina AV et al Cancer Res 2006 using GPL96 | 17079448 | ER negative vs. ER positive |
| Ivshina AV et al Cancer Res 2006 using GPL96 | 17079448 | no recurrence vs. recurrence |
| Ivshina AV et al Cancer Res 2006 using GPL96 | 17079448 | node negative vs. node positive |
| Ivshina AV et al Cancer Res 2006 using GPL96 | 17079448 | p53 wild type vs. p53 mutant |
| Ivshina AV et al Cancer Res 2006 using GPL96 | 17079448 | relapse vs. no relapse |
| Ivshina AV et al Cancer Res 2006 using GPL97 | 17079448 | 1-like, 2a-, 2b-, and 3-like |
| Ivshina AV et al Cancer Res 2006 using GPL97 | 17079448 | ELSTON grades 1, 2, and 3 |
| Ivshina AV et al Cancer Res 2006 using GPL97 | 17079448 | ER negative vs. ER positive |
| Ivshina AV et al Cancer Res 2006 using GPL97 | 17079448 | luminal A, luminal B, Basal, ERBB2, and normal like |
| Ivshina AV et al Cancer Res 2006 using GPL97 | 17079448 | no recurrence vs. recurrence |
| Ivshina AV et al Cancer Res 2006 using GPL97 | 17079448 | node negative vs. node positive |
| Ivshina AV et al Cancer Res 2006 using GPL97 | 17079448 | p53 wild type vs. p53 mutant |
| Ivshina AV et al Cancer Res 2006 using GPL97 | 17079448 | relapse vs. no relapse |
| Jones C et al Cancer Res 2004 | 15126339 | luminal breast cells and myoepithelial breast cells |
| Julka PK et al Br J Cancer 2008 using GPL887 | 18382427 | ER negative vs. ER positive |
| Julka PK et al Br J Cancer 2008 using GPL887 | 18382427 | HER2 negative vs. HER2 positive |
| Julka PK et al Br J Cancer 2008 using GPL887 | 18382427 | luminal A, luminal B, basal, HER2, and normal like |
| Julka PK et al Br J Cancer 2008 using GPL887 | 18382427 | poorly differentiated vs. moderately differentiated |
| Julka PK et al Br J Cancer 2008 using GPL887 | 18382427 | PR negative vs. PR positive |
| Julka PK et al Br J Cancer 2008 using GPL887 | 18382427 | TNM stages T3 and T4 |
| Kang Y et al Cancer Cell 2003 | 12842083 | control , bone metastasis, and adrenal gland metastasis |
| Kang Y et al Cancer Cell 2003 | 12842083 | highly metastasis phenotype vs. weakly metastasis phenotype |
| Kreike B et al Clin Cancer Res 2006 | 17020974 | primary tumors without recurrence vs. recurrences |
| Lamb J et al Science 2006 | 17008526 | 17-allylamino-geldanamycin effects to breast cancer cell lines |
| Lamb J et al Science 2006 | 17008526 | control and 17-allylamino-geldanamycin |
| Lamb J et al Science 2006 | 17008526 | control and 17-dimethylamino-geldanamycin |
| Lamb J et al Science 2006 | 17008526 | control and Alpha Estradiol |
| Lamb J et al Science 2006 | 17008526 | control and Arachidonic |
| Lamb J et al Science 2006 | 17008526 | control and Geldanamycin |
| Lamb J et al Science 2006 | 17008526 | control vs. Haloperidol |
| Lamb J et al Science 2006 | 17008526 | control vs. thioridazine |
| Lamb J et al Science 2006 | 17008526 | control, Fulvestrant 10nM, and Fulvestrant 1uM |
| Lamb J et al Science 2006 | 17008526 | estradiol and control |
| Lamb J et al Science 2006 | 17008526 | genistein 1uM and Genistein 10uM |
| Lamb J et al Science 2006 | 17008526 | genistein vs. control |
| Lamb J et al Science 2006 | 17008526 | vorinostat vs. control |
| Li Z et al Cancer Cell 2007 | 18068631 | Eph EN tumor vs. Eph EN DN tumor |
| Li Z et al Cancer Cell 2007 | 18068631 | ETV6 NTRK3 transduced NIH 3T3 cells |
| Li Z et al Cancer Cell 2007 | 18068631 | WT mammary gland, FACS sorted tumor epithelial cells |
| Lin CY et al Breast Cancer Res 2007 | 17428314 | time series treatment by ER beta and E2 |
| Loi S et al BMC Genomics 2008 using GPL570 | 18498629 | grade 1, 2, and 3 |
| Loi S et al BMC Genomics 2008 using GPL570 | 18498629 | PR negative ER positive vs. PR positive ER positive |
| Loi S et al BMC Genomics 2008 using GPL570 | 18498629 | tumor size comparison in GPL570 |
| Loi S et al BMC Genomics 2008 using GPL96 | 18498629 | grade 1, 2, and 3 |
| Loi S et al BMC Genomics 2008 using GPL96 | 18498629 | node negative vs. node positive |
| Loi S et al BMC Genomics 2008 using GPL96 | 18498629 | RFS negative vs. RFS positive |
| Loi S et al BMC Genomics 2008 using GPL96 | 18498629 | Tamoxifen treated vs. Untreated |
| Loi S et al BMC Genomics 2008 using GPL96 | 18498629 | tumor size in GPL96 |
| Loi S et al BMC Genomics 2008 using GPL97 | 18498629 | ER negative vs. ER positive |
| Loi S et al BMC Genomics 2008 using GPL97 | 18498629 | grade 1, 2, and 3 |
| Loi S et al BMC Genomics 2008 using GPL97 | 18498629 | node negative vs. node positive |
| Loi S et al BMC Genomics 2008 using GPL97 | 18498629 | RFS negative vs. RFS positive |
| Loi S et al BMC Genomics 2008 using GPL97 | 18498629 | tamoxifen untreated vs. tamoxifen treated |
| Loi S et al BMC Genomics 2008 using GPL96 | 18498629 | ER negative vs. ER positive |
| Lu X et al Breast Cancer Res Treat 2008 | 18297396 | breast cancer grades 1,2,3 |
| Lu X et al Breast Cancer Res Treat 2008 | 18297396 | ductal breast cancer, lobular breast cancer, and mixed |
| Lu X et al Breast Cancer Res Treat 2008 | 18297396 | ER negative vs. ER positive |
| Lu X et al Breast Cancer Res Treat 2008 | 18297396 | HER2 negative vs. HER2 positive |
| Lu X et al Breast Cancer Res Treat 2008 | 18297396 | LVI negative vs. LVI positive |
| Lu X et al Breast Cancer Res Treat 2008 | 18297396 | node negative vs. node positive |
| Lu X et al Breast Cancer Res Treat 2008 | 18297396 | tumor size comparisons |
| Ma XJ et al Cancer Cell 2004 | 15193263 | grade 1, 2, and 3 |
| Ma XJ et al Cancer Cell 2004 | 15193263 | no recurrence vs. recurrence |
| Ma XJ et al Cancer Cell 2004 | 15193263 | PR negative vs. PR positive |
| Ma XJ et al PNAS 2003 | 12714683 | atypical ductal hyperplasia and ductal carcinoma progression |
| Ma XJ et al PNAS 2003 | 12714683 | breast cancer grades I, II, III |
| Ma XJ et al PNAS 2003 | 12714683 | ER+ and ER- breast cancer |
| Ma XJ et al PNAS 2003 | 12714683 | Her2+ and Her2- breast cancer |
| Ma XJ et al PNAS 2003 | 12714683 | nod+ vs. nod- |
| Ma XJ et al PNAS 2003 | 12714683 | PR+ and PR- breast cancer |
| Miller LD et al PNAS 2005 using GPL96 | 16141321 | breast cancer grade 1, 2, and 3 |
| Miller LD et al PNAS 2005 using GPL96 | 16141321 | ER negative vs. ER positive |
| Miller LD et al PNAS 2005 using GPL96 | 16141321 | node negative vs. node positive |
| Miller LD et al PNAS 2005 using GPL96 | 16141321 | p53 wild type vs. p53 mutant |
| Miller LD et al PNAS 2005 using GPL96 | 16141321 | PR negative vs. PR positive |
| Miller LD et al PNAS 2005 using GPL97 | 16141321 | breast cancer grades 1, 2, and 3 |
| Miller LD et al PNAS 2005 using GPL97 | 16141321 | ER negative vs. ER positive |
| Miller LD et al PNAS 2005 using GPL97 | 16141321 | node negative vs. node positive |
| Miller LD et al PNAS 2005 using GPL97 | 16141321 | p53 wild type vs. p53 mutant |
| Miller LD et al PNAS 2005 using GPL97 | 16141321 | PR negative vs. PR positive |
| Oh DS et al J Clin Oncol 2006 using GPL1390 | 16505416 | 45 to 65 vs. more than 65 |
| Oh DS et al J Clin Oncol 2006 using GPL1390 | 16505416 | any size and direct extension to chest wall or skin |
| Oh DS et al J Clin Oncol 2006 using GPL1390 | 16505416 | grade 1, 2, and 3 |
| Oh DS et al J Clin Oncol 2006 using GPL1390 | 16505416 | no relapse vs. relapse or die of disease |
| Oh DS et al J Clin Oncol 2006 using GPL1708 | 16505416 | MCF7 Estrogen deprivation, transfected with GATA3 |
| Oh DS et al J Clin Oncol 2006 using GPL887 | 16505416 | any size and direct extension to chest wall or skin |
| Oh DS et al J Clin Oncol 2006 using GPL887 | 16505416 | ER negative vs. ER positive |
| Oh DS et al J Clin Oncol 2006 using GPL887 | 16505416 | grade 1, 2, and 3 |
| Oh DS et al J Clin Oncol 2006 using GPL887 | 16505416 | no relapse vs. relapse or die of disease |
| Oh DS et al J Clin Oncol 2006 using GPL887 | 16505416 | Node negative vs. Node positive |
| Oh DS et al J Clin Oncol 2006 using GPL1390 | 16505416 | ER negative vs. ER positive |
| Oh DS et al J Clin Oncol 2006 using GPL885 | 16505416 | ER negative vs. ER positive |
| Oh DS et al J Clin Oncol 2006 using GPL887 | 16505416 | less than 45, 45 to 65, older than 65 |
| Perou CM et al PNAS 1999 | 10430922 | HMEC, abnormal HMEC, and breast cancer. |
| Ramaswamy S et al PNAS 2001 | 11742071 | breast cancer tissues vs. normal breast tissues |
| Smirnov DA et al Cancer Res 2006 | 16540638 | tumor tissues vs. normal tissues |
| Sotiriou C et al J Natl Cancer Inst 2006 | 16478745 | ER negative vs. ER positive |
| Sotiriou C et al J Natl Cancer Inst 2006 | 16478745 | grades 1, 2, and 3 |
| Sotiriou C et al J Natl Cancer Inst 2006 | 16478745 | tamoxifen treated primary breast cancer |
| Sotiriou C et al J Natl Cancer Inst 2006 | 16478745 | tumor size comparison |
| Sotiriou C et al PNAS 2003 | 12917485 | basal-like 1,basal-like 2, and Her-2/neu |
| Sotiriou C et al PNAS 2003 | 12917485 | ER status |
| Sotiriou C et al PNAS 2003 | 12917485 | grade status (I and III) |
| Sotiriou C et al PNAS 2003 | 12917485 | luminal-like 1, luminal-like 2, and luminal-like 3 |
| Stitziel NO et al Cancer Res2004 | 15574777 | MCF-7 CYT fraction vs. MCF-7 MEM fraction |
| Troester MA et al BMC Cancer 2006 using GPL885 | 17150101 | sham treated p53 RNAi, Dox-treated p53 RNAi cell lines |
| Turashvili G et al BMC Cancer 2007 | 17389037 | ductal invasive vs. lobular invasive breast carcinomas |
| van de Vijver MJ et al N Engl J Med 2002 | 12490681 | breast cancer metastasis |
| van de Vijver MJ et al N Engl J Med 2002 | 12490681 | ER negative breast cancer vs. ERCpositive breast cancer |
| van de Vijver MJ et al N Engl J Med 2002 | 12490681 | lymphnode status |
| van 't Veer LJ et al Nature 2002 | 11823860 | distant metastasis and BRCA1 germline mutations |
| Wang Y et al Lancet 2005 | 15721472 | breast cancers without relapse vs. Breast cancers with relapse |
| Wang Y et al Lancet 2005 | 15721472 | ER positive breast cancers vs. ER negative breast cancers |
| Weigelt B et al Cancer Res 2005 | 16230372 | breast cancer subtype comparisons |
| Weigelt B et al Cancer Res 2005 | 16230372 | breast cancer subtype Luminal and Normal Breast-like |
| Weigelt B et al Cancer Res 2005 | 16230372 | HER2+ subtype, Basal-like, Normal Breast-like |
| Weigelt B et al PNAS 2003 | 14665696 | ER-alpha-negative vs. ER-alpha-positive |
| West M et al PNAS 2001 | 11562467 | ER and lymph node status |
| West M et al PNAS 2001 | 11562467 | ER+ tumors vs. ER- tumors |
| White SL et al Br J Cancer 2004 | 14710226 | C3.6 (EGF), C3.6 (Hrgb1), HB4a (EGF), and HB4a |
| Yau C et al Breast Cancer Res 2007 | 17850661 | node negative vs. node positive |
| Yu K et al Clin Cancer Res 2006 | 16740749 | ER negative vs. ER positive |
| Yu K et al Clin Cancer Res 2006 | 16740749 | grades 1, 2, and 3 |
| Yu K et al Clin Cancer Res 2006 | 16740749 | node negative vs. node positive |
| Yu K et al Clin Cancer Res 2006 | 16740749 | PR negative vs. PR positive |
| Zhou Y et al BMC Cancer 2007 | 17407600 | no recurrence vs. recurrence |
