# Supplementary material for: A Data Similarity-Based Strategy for Meta-analysis of Transcriptional Profiles in Cancer
Source: PLoS One. 2013 Jan 29;8(1):e54979. doi: 10.1371/journal.pone.0054979 (PMC3558433; doi:10.1371/journal.pone.0054979)
Supplement: Table S2 — List of breast cancer meta-signatures. (DOCX) [file pone.0054979.s005.docx]

**Table S2. List of breast cancer meta-signatures**

| **Signatures** | **Source** | **Method** | **Role** |
| --- | --- | --- | --- |
| BRmet50 | 11 signatures | clustering and assembling | value test |
| BRSig70 | BR70 dataset | supervised selection | positive control |
| BRSig76 | BR76 dataset | supervised selection | positive control |
| BRmet[-1042] | 10 signatures | leave-one-out clustering | cross validation in BR1042 |
| BRmet[-1095] | 10 signatures | leave-one-out clustering | cross validation in BR1095 |
| BRmet[-1128] | 10 signatures | leave-one-out clustering | cross validation in BR1128 |
| BRmet[-1141] | 10 signatures | leave-one-out clustering | cross validation in BR1141 |
| BRmet[-1405] | 10 signatures | leave-one-out clustering | cross validation in BR1405 |
| BRmet[-1414] | 10 signatures | leave-one-out clustering | cross validation in BR1414 |
| BRmet[-1552] | 10 signatures | leave-one-out clustering | cross validation in BR1552 |
| BRmet[-2411] | 10 signatures | leave-one-out clustering | cross validation in BR2411 |
| BRmet[-544] | 10 signatures | leave-one-out clustering | cross validation in BR544 |
