# Supplementary material for: A Data Similarity-Based Strategy for Meta-analysis of Transcriptional Profiles in Cancer
Source: PLoS One. 2013 Jan 29;8(1):e54979. doi: 10.1371/journal.pone.0054979 (PMC3558433; doi:10.1371/journal.pone.0054979)
Supplement: Table S3 — Annotation of genes in BRmet50. (DOCX) [file pone.0054979.s006.docx]

**Table S3. Annotation of genes in BRmet50.**

| Gene | Meta- | Expression | Role | Function |
| --- | --- | --- | --- | --- |
| Symbol | Direction | in Cancer | in Cancer | Category |
| UBE2C | Up* | Up[31,32] | Progression[31,32] | Cell Cycle |
| KIF2C | Up | Up[33] | Progression[6] | Cell Cycle |
| TACC3 | Up | Up[34] | Progression[34] | Cell Cycle |
| MAD2L1 | Up | Up[35] | Progression[35] | Cell Cycle |
| AURKA | Up | Up[36] | Progression[36] | Cell Cycle |
| CEP55 | Up | Up[37] | Progression[38] | Cell Cycle |
| CCNB1 | Up | Up[39] | Progression[40] | Cell Cycle |
| RRM2 | Up | Up[41,42] | Progression[17] | Cell Cycle |
| DLGAP5 | Up | Up[43] | Progression[43] | Cell Cycle |
| NEK2 | Up | Up[44] | Progression[45] | Cell Cycle |
| NDC80 | Up | Up[46] | NA* | Cell Cycle |
| UBE2S | Up | Up[11] | NA | Cell Cycle |
| CCNB2 | Up | Up[47] | NA | Cell Cycle |
| KIF20A | Up | Up[48] | NA | Cell Cycle |
| TRIP13 | Up | Up[49] | NA | Cell Cycle |
| CDKN3 | Up | Up[50] | NA | Cell Cycle |
| RAD51 | Up | Up[51] | Progression[51] | DNA Replication |
| KPNA2 | Up | Up[13] | Progression[13] | DNA Replication |
| TYMS | Up | Up[52] | NA | DNA Replication |
| CDT1 | Up | Up[53] | NA | DNA Replication |
| **Table S3. Annotation of genes in BRmet50 (cont).** | | | | |
| FEN1 | Up | Up[54] | NA | DNA Replication |
| RFC4 | Up | Up[55] | NA | DNA Replication |
| EZH2 | Up | Up[56] | Progression[56] | Proliferation |
| DDX39 | Up | Up[57] | Progression[57] | Proliferation |
| GTPBP4 | Up | Down[20] | Suppressor[20] | Proliferation |
| CCT5 | Up | Up[58] | NA | Protein Folding |
| HJURP | Up | NA | NA | Cell Cycle |
| SPAG5 | Up | NA | NA | Cell Cycle |
| KIF4A | Up | NA | NA | Cell Cycle |
| PRC1 | Up | NA | NA | Cell Cycle |
| KIF23 | Up | NA | NA | Cell Cycle |
| NUSAP1 | Up | NA | NA | Cell Cycle |
| CENPN | Up | NA | NA | Cell Cycle |
| LRP8 | Up | NA | NA | Cell Movement |
| GMPS | Up | NA | NA | DNA Replication |
| MCM10 | Up | NA | NA | DNA Replication |
| CDC45L | Up | NA | NA | DNA Replication |
| GARS | Up | NA | NA | Proliferation |
| C1orf106 | Up | NA | NA | NA |
| BTG2 | Down* | Down[21] | Suppressor[21] | Anti-Proliferation |
| SCUBE2 | Down | Down[22] | Suppressor[22] | Anti-Proliferation |
| OGN | Down | Down[59] | NA | Cellular Assembly |
| **Table S3. Annotation of genes in BRmet50 (cont).** | | | | |
| SH3BGRL | Down | Down[60] | NA | Thioredoxin Fold Proteins |
| COL14A1 | Down | NA | NA | Cellular Assembly |
| SPARCL1 | Down | NA | NA | Cellular Assembly |
| RAI2 | Down | NA | NA | Proliferation |
| KIF13B | Down | NA | NA | Cell Movement |
| QDPR | Down | NA | NA | Aamino Acid and Oxidation |
| ALDH3A2 | Down | NA | NA | Lipid Oxidoreductase Activity |
| CIRBP | Down | NA | NA | mRNA Stabilization |

*****Up for up-regulation; Down for down-regulation; NA for not available.

Meta-direction: concordant expression direction of the BRmet50 genes.
