# Supplementary material for: A Data Similarity-Based Strategy for Meta-analysis of Transcriptional Profiles in Cancer
Source: PLoS One. 2013 Jan 29;8(1):e54979. doi: 10.1371/journal.pone.0054979 (PMC3558433; doi:10.1371/journal.pone.0054979)
Supplement: Table S4 — Hazard ratio risks and log-rank tests in BR1141. (DOCX) [file pone.0054979.s007.docx]

**Table S4. Hazard ratio risks and log-rank tests in BR1141**

|  | **BRmet50 Control** | | **BRsig70** |  | **BRsig76** | |
| --- | --- | --- | --- | --- | --- | --- |
| Tumor features | **HR (95% CI)** | **HR P** | **HR (95% CI)** | **HR P** | **HR (95% CI)** | **HR P** |
| **Tumor size** |  |  |  |  |  |  |
| **T1** | 2.5 (1.2-5.1) | 0.014 | 1.5 (0.6-3.7) | 0.386 | 1.0 (0.5-2.1) | 0.942 |
| **T2** | 2.0 (1.2-3.3) | 0.009 | 1.8 (0.9-3.8) | 0.113 | 0.7 (0.4-1.2) | 0.209 |
| **Lymph node involvement** |  |  |  |  |  |  |
| **No** | 2.2 (1.3-3.7) | 0.003 | 1.6 (0.8-3.0) | 0.193 | 0.8 (0.5-1.4) | 0.511 |
| **Yes** | 2.8 (1.4-5.6) | 0.004 | 2.8 (0.8-9.3) | 0.089 | 0.6 (0.3-1.4) | 0.245 |
| **Tamoxifen treatment** |  |  |  |  |  |  |
| **No** | 2.7 (1.3-5.5) | 0.007 | 2.1 (1.0-4.6) | 0.063 | 1.1 (0.5-2.0) | 0.869 |
| **Yes** | 2.6 (1.5-4.6) | 0.001 | 1.7 (0.7-4.0) | 0.230 | 0.6 (0.3-1.0) | 0.041 |
| **Differentiation** |  |  |  |  |  |  |
| **Good** | 2.5 (0.8-7.4) | 0.105 | 2.4 (0.8-7.2) | 0.121 | 1.3 (0.4-3.8) | 0.682 |
| **Intermediate** | 2.9 (1.7-5.0) | <0.001 | 1.6 (0.8-3.4) | 0.219 | 0.7 (0.4-1.2) | 0.194 |
| **Poor** | 1.3 (0.5-3.1) | 0.602 | 0.2 (0-1.8) | 0.172 | 0.5 (0.2-1.1) | 0.086 |
| **ER status** |  |  |  |  |  |  |
| **Negative** | 1.9 (0.6-6.6) | 0.310 | 2.2 (0.3-16.3) | 0.456 | 0.9 (0.3-2.3) | 0.782 |
| **Positive** | 2.5 (1.6-4.0) | <0.001 | 1.8 (1.0-3.3) | 0.050 | 0.7 (0.4-1.1) | 0.103 |
