# Supplementary material for: A Data Similarity-Based Strategy for Meta-analysis of Transcriptional Profiles in Cancer
Source: PLoS One. 2013 Jan 29;8(1):e54979. doi: 10.1371/journal.pone.0054979 (PMC3558433; doi:10.1371/journal.pone.0054979)
Supplement: Table S5 — Comparison of signatures with common clinicopathologic factors by univariate hazard ratio model. (DOCX) [file pone.0054979.s008.docx]

**Table S5. Comparison of signatures with common clinicopathologic factors by univariate hazard ratio model**

| **Classifiers** |  | **BR1042** | **BR1095** | **BR1128** | **BR1141** | **GSE7390** |
| --- | --- | --- | --- | --- | --- | --- |
| **BRmet50*** | HR (95% CI) | 2.8 (1.4 - 5.5) | 2.2 (1.4 - 3.3) | 2.8 (1.5 - 4.9) | 2.2 (1.5 - 3.3) | 1.7 (1.1 -2.5) |
|  | p-value | **<0.01** | **<0.01** | **<0.01** | **<0.01** | **0.03** |
| **BRmet50 control** | HR (95% CI) | 2.0 (1.0 - 3.8) | 2.2 (1.4 - 3.3) | 2.8 (1.5 - 4.9) | 2.4 (1.6 - 3.6) | ND |
|  | p-value | **0.03** | **<0.001** | **<0.001** | **<0.001** | **ND** |
| **BRSig70** | HR (95% CI) | 2.0 (0.9 - 4.2) | 1.9 (1.3 - 3.0) | 1.9 (1.1 - 3.3) | 1.9 (1.1 - 3.4) | 1.1 (0.8 - 1.7) |
|  | p-value | 0.07 | <0.01 | 0.01 | 0.03 | 0.52 |
| **BRSig76** | HR (95% CI) | 1.1 (0.6 - 2.2) | 1.9 (1.2 - 3.0) | 2.0 (1.1 - 3.5) | 0.7 (0.5 - 1.1) | 1.4 (1.0 - 2.5) |
|  | p-value | 0.7 | <0.01 | 0.02 | 0.16 | 0.06 |
| **NPI**** | HR (95% CI) | 1.6 (1.1 - 2.3) | 1.7 (1.3 - 2.1) | 2.2 (1.6 - 2.9) | 1.4 (1.1 - 1.8) | 1.1 (0.8 - 1.5) |
|  | p-value | 0.02 | <0.01 | <0.01 | <0.01 | 0.46 |
| **Size** | HR (95% CI) | 1.6 (0.8 - 3.0) | 2.5 (1.6 - 3.7) | 3.2 (1.8 - 5.5) | 2.1 (1.4 - 3.3) | 1.2 (0.8 - 1.8) |
|  | p-value | 0.15 | <0.01 | <0.01 | <0.01 | 0.36 |
| **Grade** | HR (95% CI) | 1.5 (1.0 - 2.3) | 1.8 (1.3 - 2.4) | 2.0 (1.4 - 3.0) | 1.3 (1.0 - 1.7) | 1.1 (0.8 - 1.4) |
|  | p-value | 0.07 | <0.01 | <0.01 | 0.08 | 0.69 |
| **Lymph node** | HR (95% CI) | 1.0 (1.0 - 1.0) | 2.2 (1.4 - 3.3) | 4.0 (2.3 - 6.9) | 1.5 (1.0 - 2.4) | 1.0 (1.0 - 1.0) |
|  | p-value | 1.00 | <0.01 | <0.01 | 0.05 | 1.00 |
| **ER** | HR (95% CI) | 0.7 (0.4 - 1.4) | 0.9 (0.5 - 1.6) | 1.3 (0.5 - 3.0) | 0.8 (0.5 - 1.3) | 0.8 (0.5 - 1.1) |
|  | p-value | 0.36 | 0.76 | 0.59 | 0.31 | 0.22 |
| **Age** | HR (95% CI) | 1.0 (1.0 - 1.0) | 1.0 (1.0 - 1.0) | 1.0 (1.0 - 1.0) | 1.0 (1.0 - 1.0) | 1.0 (1.0 - 1.0) |
|  | p-value | 0.45 | 0.72 | 0.73 | 0.89 | 0.42 |

***BRmet50 control signatures were tested by three BR datasets, and BRmet50 was examined in**

**all breast cancer datasets.**

****NPI:** Nottingham Prognostic Index.
