# Supplementary material for: A Data Similarity-Based Strategy for Meta-analysis of Transcriptional Profiles in Cancer
Source: PLoS One. 2013 Jan 29;8(1):e54979. doi: 10.1371/journal.pone.0054979 (PMC3558433; doi:10.1371/journal.pone.0054979)
Supplement: Table S6 — Multivariate analysis of relapse risk among patients with breast cancer. (DOCX) [file pone.0054979.s009.docx]

**Table S6. Multivariate analysis of relapse risk among patients with breast cancer**

|  | **BRmet50 control** | | **BRSig70** | | **BRSig76** | |
| --- | --- | --- | --- | --- | --- | --- |
|  | **HR (95% CI)** | **HR P** | **HR (95% CI)** | **HR P** | **HR (95% CI)** | **HR P** |
| **BR1042** | 2.3 (1.0 - 5.2) | 0.04 | 1.7 (0.7 - 3.9) | 0.23 | 0.8 (0.4 - 1.7) | 0.54 |
| **BR1095** | 1.8 (1.1 - 2.9) | 0.02 | 1.5 (0.9 - 2.5) | 0.16 | 1.3 (0.8 - 2.2) | 0.26 |
| **BR1128** | 2.0 (1.0 - 3.9) | 0.03 | 1.4 (0.8 - 2.7) | 0.27 | 1.2 (0.6 - 2.3) | 0.49 |
| **BR1141** | 2.5 (1.6 - 3.9) | <0.01 | 1.6 (0.9 - 2.9) | 0.13 | 0.6 (0.4 - 1.0) | 0.05 |
| **GSE7390** | ND | ND | 1.1 (0.6 - 2.0) | 0.76 | 2.0 (1.1 - 3.3) | 0.03 |

HR and HR P-values of signature effect adjusted by age, grade, tumor size, LN, ER, and NPI.

ND: not done.
