# Supplementary material for: A Data Similarity-Based Strategy for Meta-analysis of Transcriptional Profiles in Cancer
Source: PLoS One. 2013 Jan 29;8(1):e54979. doi: 10.1371/journal.pone.0054979 (PMC3558433; doi:10.1371/journal.pone.0054979)
Supplement: Methods S1 — Supplemental methods. (DOC) [file pone.0054979.s010.doc]

**A data similarity based strategy for meta-analysis of transcriptional profiles in cancer**

Qingchao Qiu, Pengcheng Lu, Yuzhu Xiang, Yu Shyr, Xi Chen, Brian David Lehmann, Daniel Joseph Viox, Alfred L George Jr. and Yajun Yi

**SUPPLEMENTAL METHODS**

**EXALT analysis**

We previously described EXALT as a novel analytical system that can compare gene-expression signatures across studies without limitations imposed by different technological platforms, different laboratories, or different species [1]. Rather than compare raw data, EXALT implements a search paradigm that matches gene-expression signatures deduced from preprocessed (i.e., normalized and background subtracted) data like those deposited in the Gene-expression Omnibus (GEO) database. Because of this feature, EXALT can compare data generated by any platform and is independent of the methods used in the initial data processing. The output from EXALT provides similarity scores and statistical confidence levels for each signature match allowing a rapid perusal of relationships between the query data and the entries in a database of signatures from other experiments. One database (HuCaSigDB) that was used extensively in this study holds published transcriptional profiling studies of human cancer (see below).

**Signature extraction and database**

We processed 223 published breast cancer gene-expression profiling datasets from 56 published breast cancer studies to enable extraction of expression signatures (Supplemental Table S1). We used a four-step process to extract gene-expression signatures from individual data sets, which we described previously [1]. First, data were formatted into a common data type. Second, we tested each gene for significant differential expression by comparing two groups of samples and calculating a Student’s t-statistic. The significant gene *p*-value (false positive rate) determined for each significant gene was adjusted by the false discovery rate (FDR) method using q-values [2]. Third, a list of significant genes with Q-values ≤ 0.2 was generated, and then the reciprocal logarithms of the Q-values (–log[Q- value]) were calculated to get the Q-scores. Finally, gene-expression signatures were generated in the form of a list of “triplets”, each defined as a gene symbol – direction code – Q-score. The direction code was determined by the relative difference in expression between two group means and could have one of three values (U, up; D, down; X, uncertain). Signatures were stored in a relational database (HuCaSigDB) linked with clinical outcome data and experimental features from the original studies. Thus, a gene expression signature as defined by EXALT is a set of significant genes with their corresponding statistical scores and direction codes. In essence, a signature (or group of signatures) represents a statistically validated ‘fingerprint’ associated with a biological observation made from a gene expression experiment.

**Determination of signature similarity**

In the iterative EXALT analysis, each query signature was compared with every subject signature in HuCaSigDB using an algorithm we described previously [1]. For each pair of query and subject signatures with lengths L_q_ and L_s_, a total identity score (TIS) was computed in three steps. First, the signatures were aligned by matching gene symbols. Then the direction codes (U, D, or X) for matching genes were determined to be concordant (i.e., U-U, or D-D), discordant (i.e., U-D), or uncertain (i.e. presence of direction code X in either query or subject). Next, the Q scores were summed separately for concordant and discordant matches to give a positive identity score (PIS) and a negative identity score (NIS), respectively, by the formulas:

PIS = ∑(S_iq_ + S_is_), i = 1,…N

NIS = – ∑(S_jq_ + S_js_), j = 1,…M

where N and M are numbers of concordant and discordant matches, respectively, and S_iq_ and S_is_ (S_jq_ and S_js_) are Q-scores for the i^th^ concordant (j^th^ discordant) match in the query and subject signatures. The NIS score was assigned a negative value because of its opposite direction from PIS scores. Matches with at least one direction code of X and all non-matching genes were excluded from the identity score calculations. Finally, the total identity score (TIS) was computed as the absolute value of the sum of the PIS and NIS divided by the sum of signature lengths (L_q_ + L_s_) using: TIS = |PIS + NIS| / (L_q_ + L_s_).

**Defining significance level**

We performed simulations to determine the statistical significance of the TIS values. We generated 1000 random query signatures and computed the TIS between each query signature and each subject signature in HuCaSigDB. The random query signatures had similar properties (length distribution, gene symbol frequency, uniqueness) to those of the actual data. The results suggested that the TIS score correlated well with query signature length. To adjust for the influence of query signature length, we derived the mean and standard deviation (SD) of the TIS as functions of query length and then normalized the TIS by converting it to its Z-score using the formula: Z_tis_-score = (TIS – mean)/SD, where mean and SD are functions of query length. This enabled us to generate an empirical distribution of Z_tis_-scores. For a real query, we followed the same procedure to calculate the Z_TIS_-score, and compared it with the empirical distribution to estimate its corresponding query *p*-value. A query is statistically significant if its *p*-value is ≤ 0.01.

**Identification of meta-signature BRmet50**

From the initial signature clusters, a single cluster was identified for its large group of signatures with common clinical phenotypes related to cancer prognosis. This cluster consisted of 11 metastasis-related signatures (Table 1). Ten out of 11 signatures shared significant similarity with the same query signature (Sig2411 in Table 1) derived from a comparison between the non-metastasis and metastasis sample groups in breast cancer [3]. Furthermore, all 11 signatures in the cluster were related to known metastasis risk factors including high-grade tumors, ER-negative status, basal-like cell type, and cancer relapse (Table 1).

To identify a recurrent and concordant gene-expression pattern in the metastatic signature cluster, we assembled the 11 signatures representing 2,034 tumor samples into a single meta-signature designated as BRmet. The genes in BRmet were ranked based on their recurrent frequencies and differential expression directions among all 11 signatures. Fifty genes (BRmet50) were present in all 11 signatures and had concordant expression vectors (annotation for these 50 genes is provided in Supplemental Table S3). Only five genes in BRmet50 overlapped with BRsig70 and two were found to be in common with BRsig76, suggesting that BRmet50 was a distinct signature. Among the 50 genes, 39 were up-regulated while the other 11 genes exhibited lower expression in aggressive tumors. Because BRmet50 was deduced from a cluster of signatures comparing highly aggressive breast cancer with less aggressive one, we predicted that BRmet50 would be associated with a poor prognosis.

**Cross validation studies using a leave-one-out approach**

We retrospectively examined BRsig70, BRsig76, and BRmet50 using 21 datasets as presented in Table 2 for the prediction of disease prognosis in breast cancer subjects. Among the 21 validation datasets, 10 of them were the aforementioned datasets used for discovering BRmet50 in Table 1 and Figure 1. To examine the stability of the iterative EXALT system and to avoid over-fitting the nine datasets, we derived nine control signatures for BRmet50 and their nine corresponding test datasets (Table 2 and Supplemental Table S2) using a leave-one-out cross validation strategy. For instance, one signature member (Sig2411) in the cluster was left out intentionally during the iterative EXALT processes. The remaining 10 signatures comprised a new cluster that was then used to assemble a new meta-signature designated as BRmet[-2411]. The left-out signature ID (Sig2411) was used to label the meta-signature and its original source dataset. For example, BRmet[-2411] indicates that the Sig2411 was dropped out before the clustering and assembling process of BRmet[-2411], and BR2411 is its source dataset. BRmet[-2411] is a special case of BRmet50. Both BRmet50 and BRmet[-2411] share the core set of the top 50 signature genes, but BRmet[-2411] has an additional 12 genes. We repeated this procedure until each signature had been was left out once. Therefore, this repetitive cross-validation procedure produced nine test BRmet signatures (Supplemental Table S2). Nine corresponding breast cancer test datasets were also made. Two datasets (BR907 and BR1224) were dropped because they had either insufficient survival data size or sample size thus failing to meet our inclusion criteria.

The independent survival validation set included 11 GEO datasets (Table 2). During survival analyses with control signatures, we only tested the control signature (e.g., BRmet[-2411]) in its corresponding dataset (e.g., BR2411) (Supplemental Table S2). However, we retrospectively tested the BRsig70, BRsig76, and BRmet50 in all 21 breast cancer test datasets for the prediction of metastasis-free survival.

**BRmet50 performance is independent of tamoxifen treatment**

To investigate whether tamoxifen treatment had any confounding effects on the utility of BRmet50, we divided the BR1141 dataset into two groups of patients based on their exposure to tamoxifen treatment and focused our analysis on the 108 patients who never received tamoxifen [4]. We used this subgroup to evaluate whether BRmet50 was independent of the confounding effects of tamoxifen treatment. Using Spearman correlation clustering and Cox proportional-hazards models, the 108 patients were stratified by BRmet50 into two groups: 47 patients with good prognosis and 61 patients with poor prognosis. The association between BRmet50 and relapse outcome in these 108 patients was significant (hazard ratio = 2.6, *p* < 0.005, Table 4). In contrast, neither BRsig70 nor BRsig76 was able to successfully divide the 108 patients into two prognostic groups.

**Annotation of BRmet50 genes**

The BRmet50 gene list includes genes involved in maintaining the cell cycle (23 genes), DNA replication (9 genes), proliferation (7 genes), and cellular motility/assembly (5 genes). These functional processes contribute to tumorigenesis, cancer progression, and metastasis. More than half of the BRmet50 genes relate to cell growth (DNA replication, cell cycle, and proliferation), which is a known component of breast cancer prognostic signatures [5]. The annotated results indicate that the BRmet50 gene meta-directions and functions correlate with their roles in cancer progression and metastasis. For example, up-regulated genes are often involved in tumor progression and have functional roles in cell cycle, DNA replication, or proliferation. Tumor suppressor genes with functional roles in anti-proliferation or cellular movement/assembly are down-regulated in aggressive tumors. On the basis of these observations, we can infer the differential expression of BRmet50 genes in clinical samples and their roles in cancer pathogenesis based on their meta-directions in BRmet50 and their physiologic functions.

Although our approach uses BRmet50 as a biomarker for disease progression, these genes may have more than just prognostic value and may possibly represent common key regulators of metastatic disease. We examined the classification of the 50 genes by their functions and relevance to cancer. The resulting information can be found in Supplemental Table S3. It is not surprising that some of the BRmet50 genes are known to be involved in tumor progression. For example, 30% (15/50) of BRmet50 genes were reported previously as tumor progression genes for multiple cancer types [6-12]. They include KPNA2 and TYMS for breast cancer [13,14]; UBE2C, CCNB1, RRM2, and CCNB2 for lung cancer [15-18]; and NDC80 for prostate cancer [19]. The data support our result in Table 6 indicating that BRmet50 represents a conserved transcription profile across multiple cancer types. The differential expression directions of these 50 genes are nearly 100% concordant (i.e., up- or down-regulated at the same time) across all 11 signatures from 2,034 breast tumor samples (Table 1). A total of 39 out of the 50 genes were up-regulated, and 11 out of 50 genes were expressed at a lower level in aggressive tumors. Some of the BRmet50 gene expression profiles (29/50) have been supported by prior works (Supplemental Table S3), while gene expression directions for the other 20 have not yet been reported. Only one gene, GTPBP4, was found to have the opposite expression direction in the literature [20]. Among the 39 up-regulated genes, 14 genes have been previously reported to contribute to cancer progression and/or metastasis. Two down-regulated BRmet50 genes are known tumor suppressors (BTG2 and SCUBE2) in breast carcinomas [21,22].

**Comparison of meta-analysis methods for gene-expression profiles**

EXALT has a unique signature encoding format for summarizing transcriptional results, large signature databases, and a powerful signature search engine [1,23]. The iterative EXALT method has additional novel features. These include gathering homologous signatures for meta-analysis, consolidating heterogeneous signatures, and discovering reliable and recurrent meta-signatures for disease prognosis. These important novel features are not present in the previous EXALT program, nor can they be found in any other meta-analysis methods.

Meta-analysis of gene-expression data for the purposes of developing clinically relevant transcriptional biomarkers has many challenges that have not been previously overcome. To illustrate a new paradigm for discovering transcriptional biomarkers, we have demonstrated that a meta-analysis strategy using iterative EXALT was capable of deducing novel meta-signatures using data from multiple independent transcriptional profiling studies. This was highlighted by the identification of a 50-gene profile (BRmet50) for predicting increased risks of metastasis and poor clinical outcome in breast cancer. We subsequently showed that this signature outperformed BRsig70 and BRsig76, especially when clinical covariates were considered. Specifically, BRmet50 is capable of predicting breast cancer clinical outcome independent of tumor size, lymph-node status, tamoxifen treatment, histological grade, and ER status. Furthermore, BRmet50, not BRsig70 or BRsig76, had predictive value in lung and prostate cancer, suggesting that this meta-signature may be a marker for common and important transcriptional events shared by multiple neoplasms. These findings demonstrate the utility of iterative EXALT meta-analysis for identifying novel transcriptional biomarkers with predictive power in breast cancer and other cancers.

The success of our approach may be mainly attributable to a large sample size and our unique meta-analysis strategy. Like any other gene-expression data analysis, simple signatures are extracted from clinically defined group comparisons, but iterative EXALT has performed integrated analysis to extract simple signatures using a much larger sample size. In this study alone, a total of 223 datasets containing 10,581 tumor samples (discovery set) were included. Next, iterative EXALT carried out signature clustering to identify a signature cluster. The simple signatures in the cluster are biologically related to breast cancer prognosis and share significant data similarity, but they represent individual gene-expression profiles. To identify a recurrent and concordant gene-expression pattern, iterative EXALT assembled these 11 homologous signatures into a synthetic signature designated as the breast cancer metastasis meta-signature (BRmet50). Therefore, BRmet50 is neither a replicate of the original signatures nor simple intersections of the overlapping genes from multiple clustered signatures. Rather, it conceptually represents an expanded expression profile with inclusion of the expression directions and confidence levels of the individual signature genes.

For meta-analysis of transcriptional profiles, other computational platforms, such as L2L [24], LOLA [25], GeneSigDB [26], Oncomine [27], and Connectivity Map (CMAP) [28], have been described but have not been implemented successfully to extract meta-signatures. These methods typically analyze a limited number of data sets brought together through a prior knowledge-based search (inclusion/exclusion criteria) rather than by intrinsic data similarities. Such approaches are inadequate given that (1) they can miss valuable datasets and (2) they can include incorrect data sets, resulting in abnormal heterogeneous expression profiles. This characteristic can negatively affect the profile performance, robustness, and applicability. For example, the Connectivity Map (CMAP) is a gene-expression signature database application derived using Gene Set Enrichment Analysis (GSEA) [28,29]. As a data integration meta-analysis application, GSEA can compare or annotate input signatures using molecular knowledge (gene loci or gene signature-related pathways), but there is not an accompanying expression signature database (e.g., GEO or Human Cancer) in GSEA. CMAP has a signature database that focuses exclusively on drug-treated cell line expression data from one platform using a single source. In Oncomine, meta-analysis is enabled by searching for a gene name or experimental keyword rather than by signature similarity [30]. This approach does not support signature comparisons across datasets or clustering of signatures. Other approaches for meta-analysis of transcriptional profiles have similar limitations.

References

1. Yi Y, Li C, Miller C, George AL, Jr. (2007) Strategy for encoding and comparison of gene expression signatures. Genome Biol 8: R133.

2. Rhodes DR, Barrette TR, Rubin MA, Ghosh D, Chinnaiyan AM (2002) Meta-analysis of microarrays: interstudy validation of gene expression profiles reveals pathway dysregulation in prostate cancer. Cancer Res 62: 4427-4433.

3. van d, V, He YD, van't Veer LJ, Dai H, Hart AA et al. (2002) A gene-expression signature as a predictor of survival in breast cancer. N Engl J Med 347: 1999-2009.

4. Loi S, Haibe-Kains B, Desmedt C, Wirapati P, Lallemand F et al. (2008) Predicting prognosis using molecular profiling in estrogen receptor-positive breast cancer treated with tamoxifen. BMC Genomics 9: 239.

5. Hu Z, Fan C, Oh DS, Marron JS, He X et al. (2006) The molecular portraits of breast tumors are conserved across microarray platforms. BMC Genomics 7: 96.

6. Nakamura Y, Tanaka F, Haraguchi N, Mimori K, Matsumoto T et al. (2007) Clinicopathological and biological significance of mitotic centromere-associated kinesin overexpression in human gastric cancer. Br J Cancer 97: 543-549.

7. Li GQ, Li H, Zhang HF (2003) Mad2 and p53 expression profiles in colorectal cancer and its clinical significance. World J Gastroenterol 9: 1972-1975.

8. Fluge O, Gravdal K, Carlsen E, Vonen B, Kjellevold K et al. (2009) Expression of EZH2 and Ki-67 in colorectal cancer and associations with treatment response and prognosis. Br J Cancer 101: 1282-1289.

9. Samaras V, Stamatelli A, Samaras E, Arnaoutoglou C, Arnaoutoglou M et al. (2009) Comparative immunohistochemical analysis of aurora-A and aurora-B expression in human glioblastomas. Associations with proliferative activity and clinicopathological features. Pathol Res Pract 205: 765-773.

10. de RA, Assie G, Rickman DS, Tissier F, Groussin L et al. (2009) Gene expression profiling reveals a new classification of adrenocortical tumors and identifies molecular predictors of malignancy and survival. J Clin Oncol 27: 1108-1115.

11. Chen MF, Lee KD, Lu MS, Chen CC, Hsieh MJ et al. (2009) The predictive role of E2-EPF ubiquitin carrier protein in esophageal squamous cell carcinoma. J Mol Med 87: 307-320.

12. Petropoulou C, Kotantaki P, Karamitros D, Taraviras S (2008) Cdt1 and Geminin in cancer: markers or triggers of malignant transformation? Front Biosci 13: 4485-4494.

13. Dankof A, Fritzsche FR, Dahl E, Pahl S, Wild P et al. (2007) KPNA2 protein expression in invasive breast carcinoma and matched peritumoral ductal carcinoma in situ. Virchows Arch 451: 877-881.

14. Miyashita M, Yoshimura H, Hatta K, Tachibana S, Kubota M et al. (2009) [Clinical significance of intratumoral TS levels and DPD activity in breast cancer]. Gan To Kagaku Ryoho 36: 407-411.

15. Kadara H, Lacroix L, Behrens C, Solis L, Gu X et al. (2009) Identification of gene signatures and molecular markers for human lung cancer prognosis using an in vitro lung carcinogenesis system. Cancer Prev Res (Phila Pa) 2: 702-711.

16. Cooper WA, Kohonen-Corish MR, McCaughan B, Kennedy C, Sutherland RL et al. (2009) Expression and prognostic significance of cyclin B1 and cyclin A in non-small cell lung cancer. Histopathology 55: 28-36.

17. Boukovinas I, Papadaki C, Mendez P, Taron M, Mavroudis D et al. (2008) Tumor BRCA1, RRM1 and RRM2 mRNA expression levels and clinical response to first-line gemcitabine plus docetaxel in non-small-cell lung cancer patients. PLoS One 3: e3695.

18. Stav D, Bar I, Sandbank J (2007) Usefulness of CDK5RAP3, CCNB2, and RAGE genes for the diagnosis of lung adenocarcinoma. Int J Biol Markers 22: 108-113.

19. Glinsky GV, Berezovska O, Glinskii AB (2005) Microarray analysis identifies a death-from-cancer signature predicting therapy failure in patients with multiple types of cancer. J Clin Invest 115: 1503-1521.

20. Lee H, Kim D, Dan HC, Wu EL, Gritsko TM et al. (2007) Identification and characterization of putative tumor suppressor NGB, a GTP-binding protein that interacts with the neurofibromatosis 2 protein. Mol Cell Biol 27: 2103-2119.

21. Karmakar S, Foster EA, Smith CL (2009) Estradiol downregulation of the tumor suppressor gene BTG2 requires estrogen receptor-alpha and the REA corepressor. Int J Cancer 124: 1841-1851.

22. Cheng CJ, Lin YC, Tsai MT, Chen CS, Hsieh MC et al. (2009) SCUBE2 suppresses breast tumor cell proliferation and confers a favorable prognosis in invasive breast cancer. Cancer Res 69: 3634-3641.

23. Wu J, Qiu Q, Xie L, Fullerton J, Yu J et al. (2009) Web-based interrogation of gene expression signatures using EXALT. BMC Bioinformatics 10: 420.

24. Newman JC, Weiner AM (2005) L2L: a simple tool for discovering the hidden significance in microarray expression data. Genome Biol 6: R81.

25. Cahan P, Ahmad AM, Burke H, Fu S, Lai Y et al. (2005) List of lists-annotated (LOLA): a database for annotation and comparison of published microarray gene lists. Gene 360: 78-82.

26. Culhane AC, Schwarzl T, Sultana R, Picard KC, Picard SC et al. (2010) GeneSigDB--a curated database of gene expression signatures. Nucleic Acids Res 38: D716-D725.

27. Rhodes DR, Barrette TR, Rubin MA, Ghosh D, Chinnaiyan AM (2002) Meta-analysis of microarrays: interstudy validation of gene expression profiles reveals pathway dysregulation in prostate cancer. Cancer Res 62: 4427-4433.

28. Lamb J, Crawford ED, Peck D, Modell JW, Blat IC et al. (2006) The Connectivity Map: using gene-expression signatures to connect small molecules, genes, and disease. Science 313: 1929-1935.

29. Subramanian A, Tamayo P, Mootha VK, Mukherjee S, Ebert BL et al. (2005) Gene set enrichment analysis: a knowledge-based approach for interpreting genome-wide expression profiles. Proc Natl Acad Sci U S A 102: 15545-15550.

30. Rhodes DR, Yu J, Shanker K, Deshpande N, Varambally R et al. (2004) Large-scale meta-analysis of cancer microarray data identifies common transcriptional profiles of neoplastic transformation and progression. Proc Natl Acad Sci U S A 101: 9309-9314.

31. Chen S, Chen Y, Hu C, Jing H, Cao Y et al. (2010) Association of clinicopathological features with UbcH10 expression in colorectal cancer. J Cancer Res Clin Oncol 136: 419-426.

32. Jiang L, Bao Y, Luo C, Hu G, Huang C et al. (2010) Knockdown of ubiquitin-conjugating enzyme E2C/UbcH10 expression by RNA interference inhibits glioma cell proliferation and enhances cell apoptosis in vitro. J Cancer Res Clin Oncol 136: 211-217.

33. Shimo A, Tanikawa C, Nishidate T, Lin ML, Matsuda K et al. (2008) Involvement of kinesin family member 2C/mitotic centromere-associated kinesin overexpression in mammary carcinogenesis. Cancer Sci 99: 62-70.

34. Yim EK, Tong SY, Ho EM, Bae JH, Um SJ et al. (2009) Anticancer effects on TACC3 by treatment of paclitaxel in HPV-18 positive cervical carcinoma cells. Oncol Rep 21: 549-557.

35. Zhang SH, Xu AM, Chen XF, Li DH, Sun MP et al. (2008) Clinicopathologic significance of mitotic arrest defective protein 2 overexpression in hepatocellular carcinoma. Hum Pathol 39: 1827-1834.

36. Shang X, Burlingame SM, Okcu MF, Ge N, Russell HV et al. (2009) Aurora A is a negative prognostic factor and a new therapeutic target in human neuroblastoma. Mol Cancer Ther 8: 2461-2469.

37. Inoda S, Hirohashi Y, Torigoe T, Nakatsugawa M, Kiriyama K et al. (2009) Cep55/c10orf3, a tumor antigen derived from a centrosome residing protein in breast carcinoma. J Immunother 32: 474-485.

38. Chen CH, Chien CY, Huang CC, Hwang CF, Chuang HC et al. (2009) Expression of FLJ10540 is correlated with aggressiveness of oral cavity squamous cell carcinoma by stimulating cell migration and invasion through increased FOXM1 and MMP-2 activity. Oncogene 28: 2723-2737.

39. Zheng H, Hu W, Deavers MT, Shen DY, Fu S et al. (2009) Nuclear cyclin B1 is overexpressed in low-malignant-potential ovarian tumors but not in epithelial ovarian cancer. Am J Obstet Gynecol 201: 367-6.

40. de HT, Hasselt N, Troost D, Caron H, Popovic M et al. (2008) Molecular risk stratification of medulloblastoma patients based on immunohistochemical analysis of MYC, LDHB, and CCNB1 expression. Clin Cancer Res 14: 4154-4160.

41. Zhang K, Hu S, Wu J, Chen L, Lu J et al. (2009) Overexpression of RRM2 decreases thrombspondin-1 and increases VEGF production in human cancer cells in vitro and in vivo: implication of RRM2 in angiogenesis. Mol Cancer 8: 11.

42. Duxbury MS, Ito H, Zinner MJ, Ashley SW, Whang EE (2004) RNA interference targeting the M2 subunit of ribonucleotide reductase enhances pancreatic adenocarcinoma chemosensitivity to gemcitabine. Oncogene 23: 1539-1548.

43. Zhao L, Qin LX, Ye QH, Zhu XQ, Zhang H et al. (2004) KIAA0008 gene is associated with invasive phenotype of human hepatocellular carcinoma--a functional analysis. J Cancer Res Clin Oncol 130: 719-727.

44. Szponar A, Zubakov D, Pawlak J, Jauch A, Kovacs G (2009) Three genetic developmental stages of papillary renal cell tumors: duplication of chromosome 1q marks fatal progression. Int J Cancer 124: 2071-2076.

45. Tsunoda N, Kokuryo T, Oda K, Senga T, Yokoyama Y et al. (2009) Nek2 as a novel molecular target for the treatment of breast carcinoma. Cancer Sci 100: 111-116.

46. Xiao GF, Tang HH (2008) [Expression and clinical significance of highly expressed protein in cancer (Hec 1) in human primary gallbladder carcinoma]. Xi Bao Yu Fen Zi Mian Yi Xue Za Zhi 24: 910-912.

47. Park SH, Yu GR, Kim WH, Moon WS, Kim JH et al. (2007) NF-Y-dependent cyclin B2 expression in colorectal adenocarcinoma. Clin Cancer Res 13: 858-867.

48. Taniuchi K, Nakagawa H, Nakamura T, Eguchi H, Ohigashi H et al. (2005) Down-regulation of RAB6KIFL/KIF20A, a kinesin involved with membrane trafficking of discs large homologue 5, can attenuate growth of pancreatic cancer cell. Cancer Res 65: 105-112.

49. Kang JU, Koo SH, Kwon KC, Park JW, Kim JM (2008) Gain at chromosomal region 5p15.33, containing TERT, is the most frequent genetic event in early stages of non-small cell lung cancer. Cancer Genet Cytogenet 182: 1-11.

50. Jiang R, Xia Y, Li J, Deng L, Zhao L et al. (2010) High expression levels of IKKalpha and IKKbeta are necessary for the malignant properties of liver cancer. Int J Cancer 126: 1263-1274.

51. Mitra A, Jameson C, Barbachano Y, Sanchez L, Kote-Jarai Z et al. (2009) Overexpression of RAD51 occurs in aggressive prostatic cancer. Histopathology 55: 696-704.

52. Naoe M, Ogawa Y, Morita J, Shichijo T, Fuji K et al. (2009) Expression of the fluoropyrimidine-metabolizing enzymes in bladder cancers as measured by the Danenberg tumor profile. Oncol Res 18: 153-162.

53. Seo J, Chung YS, Sharma GG, Moon E, Burack WR et al. (2005) Cdt1 transgenic mice develop lymphoblastic lymphoma in the absence of p53. Oncogene 24: 8176-8186.

54. Singh P, Yang M, Dai H, Yu D, Huang Q et al. (2008) Overexpression and hypomethylation of flap endonuclease 1 gene in breast and other cancers. Mol Cancer Res 6: 1710-1717.

55. Arai M, Kondoh N, Imazeki N, Hada A, Hatsuse K et al. (2009) The knockdown of endogenous replication factor C4 decreases the growth and enhances the chemosensitivity of hepatocellular carcinoma cells. Liver Int 29: 55-62.

56. Karanikolas BD, Figueiredo ML, Wu L (2010) Comprehensive evaluation of the role of EZH2 in the growth, invasion, and aggression of a panel of prostate cancer cell lines. Prostate 70: 675-688.

57. Sugiura T, Nagano Y, Noguchi Y (2007) DDX39, upregulated in lung squamous cell cancer, displays RNA helicase activities and promotes cancer cell growth. Cancer Biol Ther 6: 957-964.

58. Ooe A, Kato K, Noguchi S (2007) Possible involvement of CCT5, RGS3, and YKT6 genes up-regulated in p53-mutated tumors in resistance to docetaxel in human breast cancers. Breast Cancer Res Treat 101: 305-315.

59. Wang Y, Ma Y, Lu B, Xu E, Huang Q et al. (2007) Differential expression of mimecan and thioredoxin domain-containing protein 5 in colorectal adenoma and cancer: a proteomic study. Exp Biol Med (Maywood ) 232: 1152-1159.

60. Majid SM, Liss AS, You M, Bose HR (2006) The suppression of SH3BGRL is important for v-Rel-mediated transformation. Oncogene 25: 756-768.

61. Curtis C, Shah SP, Chin SF, Turashvili G, Rueda OM et al. (2012) The genomic and transcriptomic architecture of 2,000 breast tumours reveals novel subgroups. Nature 486: 346-352.

62. van ', V, Dai H, van d, V, He YD, Hart AA et al. (2002) Gene expression profiling predicts clinical outcome of breast cancer. Nature 415: 530-536.

63. Wang Y, Klijn JG, Zhang Y, Sieuwerts AM, Look MP et al. (2005) Gene-expression profiles to predict distant metastasis of lymph-node-negative primary breast cancer. Lancet 365: 671-679.

64. Paik S, Shak S, Tang G, Kim C, Baker J et al. (2004) A multigene assay to predict recurrence of tamoxifen-treated, node-negative breast cancer. N Engl J Med 351: 2817-2826.

65. Flanagan MB, Dabbs DJ, Brufsky AM, Beriwal S, Bhargava R (2008) Histopathologic variables predict Oncotype DX recurrence score. Mod Pathol 21: 1255-1261.

66. Parker JS, Mullins M, Cheang MC, Leung S, Voduc D et al. (2009) Supervised risk predictor of breast cancer based on intrinsic subtypes. J Clin Oncol 27: 1160-1167.

67. Haibe-Kains B, Desmedt C, Rothe F, Piccart M, Sotiriou C et al. (2010) A fuzzy gene expression-based computational approach improves breast cancer prognostication. Genome Biol 11: R18.

68. Loi S, Haibe-Kains B, Majjaj S, Lallemand F, Durbecq V et al. (2010) PIK3CA mutations associated with gene signature of low mTORC1 signaling and better outcomes in estrogen receptor-positive breast cancer. Proc Natl Acad Sci U S A 107: 10208-10213.

69. Sotiriou C, Wirapati P, Loi S, Harris A, Fox S et al. (2006) Gene expression profiling in breast cancer: understanding the molecular basis of histologic grade to improve prognosis. J Natl Cancer Inst 98: 262-272.
